# Melt-bearing nodules from Tenerife reveal magma reservoir diversity prior to caldera-forming eruptions

**DOI:** 10.1007/s00410-026-02302-3

**Published:** 2026-04-28

**Authors:** Emma L Horn, Rex N Taylor, Thomas M Gernon, Michael J Stock, Katherine E Schofield, Richard J. Brown, Victoria C Smith, Danielle McLean

**Affiliations:** 1 School of Ocean and Earth Science, University of Southampton, Waterfront Campus, Southampton, SO14 3ZH UK; 2 The School of Archaeology, Research Laboratory for Archaeology and the History of Art, University of Oxford, Oxford, OX1 3TG UK; 3 Discipline of Geology, School of Natural Sciences, Trinity College Dublin, Dublin 2, Ireland; 4 Department of Earth Sciences, Science Site, Durham University, Durham, DH1 3LE UK; 5 GFZ Helmholtz Centre for Geosciences, 14473 Potsdam, Germany

**Keywords:** Tenerife, Canary Islands, Crystal mush, Geochemistry, Petrology, Interstitial magma

## Abstract

Partially crystalline nodules are occasionally discovered within breccias linked to large ignimbrite-forming eruptions, providing evidence of the fragmentation of the plutonic reservoir and conduit system during such eruptions. These nodules offer valuable insights into the magmatic systems fuelling these highly explosive volcanoes. On Tenerife, crystal-rich samples containing interstitial melt are preserved in several Plinian eruption deposits spanning over ~ 1.8 million years, but the crustal architecture and interactions between magma reservoirs beneath the island remain poorly understood. This study focuses on explosively fragmented juvenile nodules from Tenerife’s pyroclastic deposits, which provide snapshots of the mush reservoir preceding caldera-forming events. Petrological, major element and trace element analyses were conducted on juvenile nodules from five major caldera-forming eruptions: Caleta (221 ka), Fasnia (312 ka), San Juan (1.50 Ma), Morteros (~ 1.70 Ma), and Gaviotas (1.84 Ma). These nodules preserve a range of crystallisation stages within Tenerife’s alkaline magmatic system, uniquely containing interstitial groundmass that existed in a supra-solidus state at the time of eruption, with an average melt content of ~ 25 vol%. Despite macro-mineralogical variability between eruptions, the juvenile nodules exhibit consistent basanite interstitial groundmass chemistry and lithologies, suggest that the mafic mush reservoir beneath Tenerife has remained both chemically and petrologically stable over ~ 1.8 million years. This study provides a new perspective on the stability of the mafic mush reservoir beneath Tenerife, highlighting its persistent role in the volcano’s magmatic plumbing system. The chemical consistency of the mush contrasts with the episodic mobilisation and more chemically diverse evolved phonolite melts, underscoring the importance of understanding mid-crustal processes leading to explosive eruptions. These findings provide evidence for a long-lived, stable mush reservoir and a new perspective on the compositional makeup of the crystal-mush reservoirs at defined points in time, enhancing our temporal understanding of ocean island volcanoes and their crustal magma mush reservoirs.

**Supplementary Information:**

The online version contains supplementary material available at 10.1007/s00410-026-02302-3.

## Introduction

Large-scale stratigraphic, petrological, and geochemical studies of ignimbrites reveal that crustal sources of major eruptions are fed by multiple geochemically and thermally distinct pulses of magma (Bachmann and Bergantz 2008a; Cashman and Giordano 2014; Ellis et al. 2014; Stock et al. 2018). However, a key challenge in the field of volcanology is determining how lower and mid-crustal magmatic processes of accumulation and ascent relate to the timing and magnitude of eruptions (Giordano and Caricchi 2022). These issues have been compounded over the last decade with the traditional model of volcanoes being underlain by discrete liquid-rich magma chambers being called into question, and emerging evidence that many volcanoes are instead underlain by large-scale mush zones, dominated by near-solidus crystal-rich material and only small, localised melt-rich regions (Cashman et al. 2017; Sparks et al. 2019). Crystal mushes represent the dominant state of magmatic storage globally, with processes of melt segregation, mechanical compaction, and disaggregation playing a fundamental role in eruption dynamics (Humphreys et al. 2025). Geophysical data indicate that these sub-volcanic mushes contain an average melt fraction of ~ 13%, based on a global compilation of seismic imaging studies (Paulatto et al. 2022). These melt fractions are rheologically immobile, underscoring the importance of the accumulation and processes within individual melt-rich chambers in the final stages of magma evolution before an eruption (Sparks and Cashman 2017). Hence, understanding the architecture and temporal development of mush zones, as well as the mechanisms of melt migration and accumulation, is key to understanding eruption frequency and styles across diverse tectonic environments (Cassidy et al. 2018; Edmonds et al. 2019; Wieser et al. 2019; Humphreys et al. 2025). Accordingly, Giordano and Caricchi (2022) emphasised the critical need for new strategies to interrogate mid-lower crustal magmatic processes in complex mush-bearing magma systems.

**Fig. 1:**
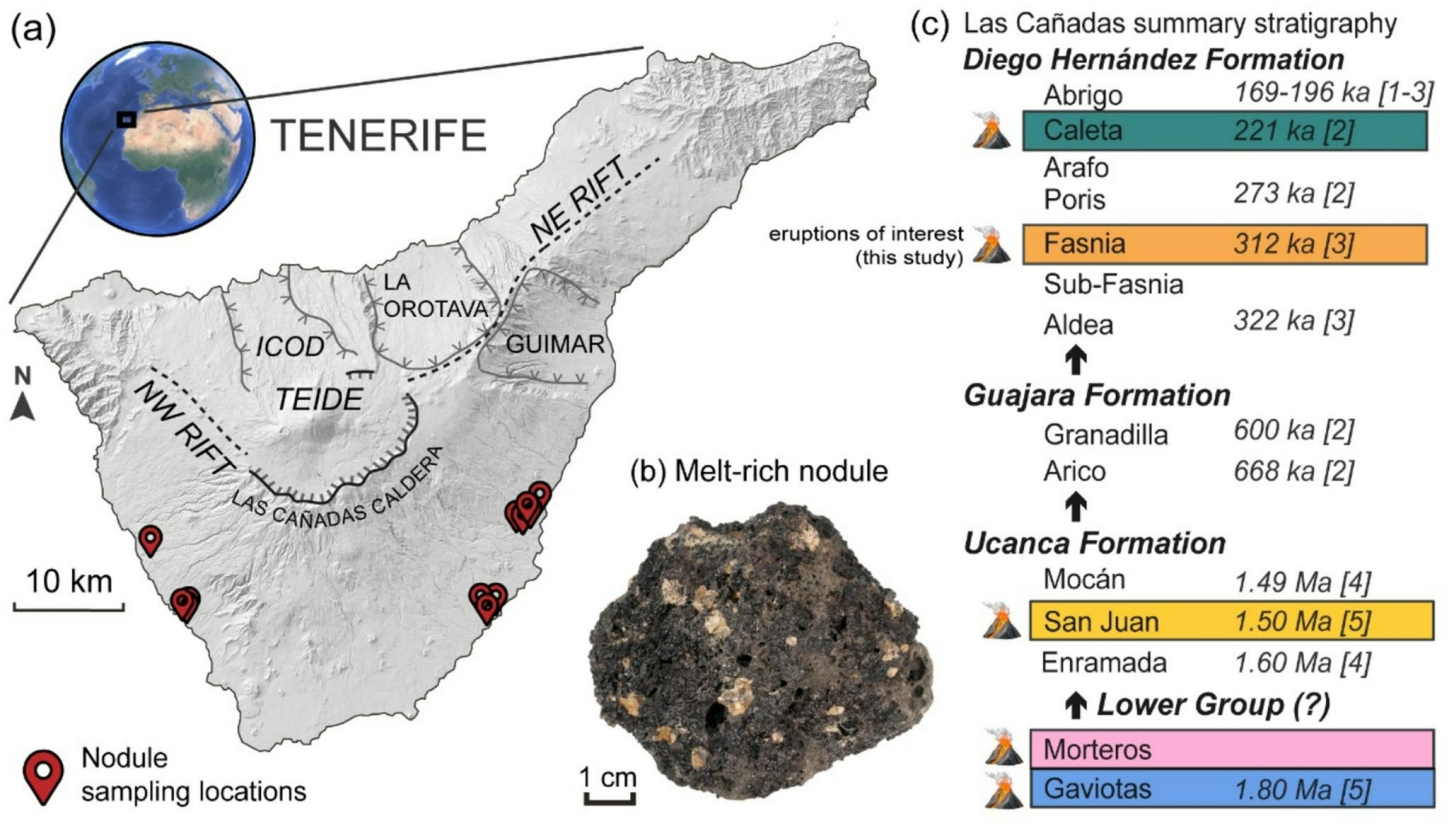
**a** Location of Tenerife and digital elevation model annotated with topographic features and juvenile nodule sampling locations. In hand specimen **b**, Photo of nodule hand specimen, the grey-vesicular regions occurring within intersitial regions between crystals are referred to as the microcrystalline groundmass or interstitial melt. **c** Shortened schematic of Las Cañadas stratigraphy highlighting the key eruptions of focus in this study, a compiled schematic is presented in Cas et al. (2022). Age references: (1) Martí et al. (1994), (2) Brown et al. (2003), (3) Edgar et al. (2007; 2017), (4) Dávila-Harris et al. (2023), (5) Huertas et al. (2002).

One approach to understanding these systems is to examine crystalline plutonic fragments which are erupted alongside magma. These are variably termed as mafic enclaves, xenoliths, cumulates, crustal fragments, and juvenile nodules. Such samples have been used to investigate the magmatic architecture and melt evolution in several different volcanic settings, including the Galapagos Archipelago, Santorini, the Lesser Antilles and the Canary Islands (Stock et al. 2012, Barker et al. 2015; Cooper et al. 2016; Masotta et al. 2016; Chamberlain et al. 2019; Holness et al. 2019; Gleeson et al. 2020; Horn et al. 2022). In the Canary Islands specifically, plutonic material is found in several volcanic deposits on La Palma, Tenerife and Lanzarote islands (Neumann et al. 2000; Barker et al. 2015; Klügel et al. 2022). Tenerife has a diverse suite of plutonic samples, including pyroxenitic, gabbroic and syenitic material (Borley et al. 1971; Scott 1976; Wolff 1987; Neumann et al. 1999, 2002; Pittari et al. 2008). The petrology of these nodules has been comprehensively studied in the context of the Fasnia eruption (Horn et al. 2022), with suites of mush-bearing nodules spanning the entire crystallisation sequence of an alkaline magmatic system, from wehrlite to monzonite. Here, we present a detailed analysis of petrological and geochemical data derived from juvenile nodules collected from four additional eruptions spanning Pleistocene Las Cañadas Edifice volcanic stratigraphy (in southern Tenerife; Fig. 1) to provide a comprehensive insight into the temporal evolution of crystal-rich magma mush reservoir. Our nodule samples include both crystalline material and quenched basanitic glass which was liquid at the time of eruption, thus preserving a quenched snapshot of a ‘live’ cumulate mush (Stock et al. 2012; Horn et al. 2022). By exploring changes in the mineral assemblage and composition of juvenile nodules erupted across ~ 1.8 Myr of Las Cañadas volcanism, and integrating our new data with existing information on nodule samples from the Fasnia eruption (Horn et al. 2022), we are able to determine the chemistry of the magma mush system over discrete points in time.

## Geological setting

Tenerife is the largest island of the Canary Islands archipelago and its oldest sub-aerial volcanism has been dated at ~ 12 Ma (Guillou et al. 2004). The early volcanic development on the island occurred in three main phases of primary shield building which formed the Anaga, Teno, and Roque del Conde massifs (11.9–3.9 Ma) and the mafic basement complex of the island (Martí et al. 1994; Guillou et al. 2004; Gottsmann et al. 2008; Longpré et al. 2009). The formation of the central Las Cañadas edifice started around ∼3.5 Ma, developing a central basanite to phonolite stratovolcano, which Upper Group was dominated by cycles of explosive, caldera-forming phonolite eruptions (Fuster 1968; Araña 1971; Ancochea et al. 1990, 1999; Martí et al. 1994). Simultaneously, basaltic activity, continued mainly along the Santiago (NE-SW) and Dorsal (NW-SE) rift zones which extend radially from the edifice and are preserved as monogenetic scoria cones and lava flows across the stratigraphy and flanks of the volcano (Carracedo et al. 2007; Dóniz et al. 2008; Kröchert and Buchner 2009; Geyer and Martí 2010; Carracedo and Perez-Torrado 2013). The most recent phase of activity is marked by the formation of the basanitic–phonolitic Pico Viejo and Pico del Teide (PV-PT) stratovolcanoes, constructed within the Las Cañadas caldera (Carracedo et al. 2011; Andújar et al. 2013). This caldera is a morphological depression formed by multiple caldera-forming eruptions (Martí et al. 1994; Ablay and Kearey 2000; Martí and Gudmundsson 2000), the last of which ended with the Abrigo eruption at ∼ 0.17 Ma (Brown et al. 2003; Edgar et al. 2007) (Table 1).

**Table 1:** Summary of the lithologies and stratigraphical information for key eruption units detailed in this study

Eruption Member/ Formation	Other named designations	Age (reference)	Eruption parameters: estimated size, volume (DRE)	Unit thickness	Compositional range (SiO_2_ wt%)
Caleta	La Caleta, Wavy Deposit, Unit M	221 ± 5 ka^a^	2.2 km^3^ (c)	fall layer < 3 m, ignimbrites ∼16 m^a^	trachyte-phonolite (58.9–60.7)
Fasnia	Lower Grey Member, Sequence III, Unit J, Deposit TEJ	289 ± 6 ka^a^;312 ± 6 ka^b, c^ (Santo ignimbrite)	VEI 5-6^b^. Total 13.3 km^3^†(c)	fallout units total: 9.42 m^b^	trachyte-phonolite (53.9–63.1)
San Juan	Taucho-playa de San Juan ignimbrites	1500 ± 30 ka^d^		5 m^d^	trachyte-phonolite (60.5–61.4)
Morteros	Lithic–rich laharic sequence	~ 1662–1840*		< 12 m^d^	phonolite (59.6–59.9)
Gaviotas	Gaviotas Ignimbrite	1840 ± 7 ka^e^		6–8 m^d^	trachyte (59.4–61.7)

A full table of the Tenerife stratigraphic nomenclature, correlation between studies and references is given in Cas et al. (2022). ^a^Brown et al. (2003), ^b^Edgar et al. (2017), ^c^Edgar et al. (2007), ^d^Dávila-Harris et al. (2009; 2023) ^e^Huertas et al. (2002). *Estimated age (explanation in text) cf., Taylor et al. (2020). †Fasnia ignimbrites are generally small in volume (0.01–0.2 km^3^ tephra). Exceptions include the Santo deposit (0.35 km^3^ tephra) and, most notably the Ravelo ignimbrite, which is the largest and most widespread deposit (~ 10 km^3^ tephra; > 1000 km^2^ dispersal area)^c^. Published whole-rock and glass geochemical data for these eruption units are available in Olin (2007), Horn et al. (2022), Dávila-Harris et al. (2023).

In this study, we examine samples from Quaternary deposits of the Las Cañadas stratovolcano complex (Fig. 1c), which are divided into an older Lower Group (3.0-1.8 Ma) and a younger Upper Group (1.57 − 0.17 Ma). The Lower Group represents a constructive phase in which the edifice evolved from the Old Basaltic Series shield to a central stratovolcano, dominated by trachytic–phonolitic effusive and phases of explosive activity and edifice building (Martí et al. 1994; Cas et al. 2022). In contrast, the Upper Group is characterised by repeated cycles of large explosive phonolitic eruptions that culminated in major caldera collapses, progressively enlarging the Las Cañadas depression (Martí et al. 1994; Cas et al. 2022). Tables 2, 3 and 4  published in Cas et al. (2022) summarise the variations in stratigraphic schemes and dating for the groups presented in previous studies.

**Table 2:** Summary of lithological, mineralogical, grain size and melt abundance data for the Caleta, San Juan, Morteros and Gaviotas juvenile nodules and lithic samples used in this study

Group	Nodule sample type	No. of samples	Mineral assemblage (major ± minor)	Major mineral abundance vol% (average)	Grain size range (mm)	Melt abundance % (average)
*Caleta Member(n = 44)*						
*1*	*Wehrlite*,* olivine clinopyroxenite*	4	**ol**,** cpx** ± opq ± bt	ol, 5–50 **(19.3)** cpx, 50–85 **(64.5)**	< 0.5–12	0–8 **(3.8)**
*1*	*Clinopyroxenite*	4	**cpx**, **opq** ± bt ± pl	cpx, 37–75 **(56.4)** opq, 1–5 **(2.7)**	1–8	1–35 **(10.6)**
*2*	*Pyroxene-hornblende gabbro*	23	**cpx**,** pl**,** hbl**, opq ± ap ± kfs ± foid* ± ol ± bt	cpx, 5–32 **(16.8)** pl, 11–35 **(26.6)** hbl, 15–43 **(27.1)**	0.5–11	0–40 **(16.7)**
*2*	*Gabbro*	1	**cpx**,** pl**, opq	cpx, 50 pl, 20	1–3	3
*2*	Hornblende rich **	9	**hbl**,** pl**,** opq** ± ap ± cpx ± kfs ± foid	hbl, 15–69 **(37.7)** pl, 10–35 **(16.6)**	0.5–11	0–53 **(21.1)**
*3*	*feldspathoid monzosyenite*,* syenite*	3	**kfs**,** pl**,** cpx** ± hbl ± foid*, opq ± ap ± bt	kfs, 30–45 **(39.6)** pl, 10–15 **(11.7)** cpx 12–15 **(13.3)**	< 0.5–7	0–8 **(2.7)**
*Fasnia Member (n = 103;* Horn et al. 2022)						
*San Juan ( n= 11)*						
*1*	*Clinopyroxenite*	2	**cpx**,** opq**	cpx, 71–77 **(74.0)** opq, 23–28 **(25.9)**	< 0.5–6	0–8 **(3.8)**
*2*	*Pyroxene-hornblende gabbro*	4	**cpx**,** pl**,** hbl**, opq ±ap ± kfs ± foid	cpx, 20–51 **(35.7)** pl, 12–54 **(31.4)** hbl, 6–27 **(14.3)**	< 0.5–4	26–6 **(14.3)**
*2*	*Gabbro*	2	**cpx**,** pl**, opq ± hbl ± ol	cpx, 63–68 **(65.8)** pl, 10–17 **(13.5)**	< 0.5 -4	23–30 **(25.8)**
*2*	*Hornblende gabbro*	2	**hbl**,** pl**, opq, cpx, ap, foid*	hbl, 41–51 **(45.9)** pl 38–41 **(39.5)**	< 0.5 -7	25–34 **(29.2)**
*3*	*Syenite*	1	**kfs**,** cpx**, pl	kfs, 81 cpx, 16	1–3	0
*Morteros (n = 20)*						
*2*	*Pyroxene-hornblende gabbro*	9	**cpx**,** pl**,** hbl**, opq ± ap ± kfs ± foid* ± ol	cpx, 0–53 **(16.3)** pl, 8–55 **(31.8)** hbl, 10–56 **(33.2)**	< 0.5–14	0–25 **(11.1)**
*2*	*Gabbro*	1	**cpx**,** pl**, opq	cpx, 63 pl, 13		20
*2*	*Hornblende gabbro*	7	**hbl**,** opq** pl, ap ± cpx ± kfs ± foid*	hbl, 40–65 **(51.6)** pl, 3–44 **(28.7)**	< 0.5–6	3–51 **(23)**
*3*	*Monzodiorite*	3	**kfs**,** pl**, cpx, hbl, opq ±ap ± bt	kfs, 0–19 **(12.3)** pl, 44–82 **(62.6)**	0.5–9	2–63 **(35.9)**
*Gaviotas (n = 20)*						
*1*	*Clinopyroxenite*	6	**cpx**,** opq**	cpx, 33–100 **(73.7)** opq, 0–65 **(26.3)**	1–8	10–33 **(16.4)**
*2*	Pyroxene-hornblende gabbro	10	cpx, pl, hbl, opq ± ap ± kfs ± foid*	cpx, 13–46 (24.2) pl, 17–51 (33.8) hbl, 5–40 (25)	< 0.5–8	1–60 **(11.1)**
*2*	*Gabbro*	1	**cpx**,** pl**, opq	cpx, 74 pl, 10	< 0.5–3	15
*2*	*Hornblende gabbro*	1	**hbl**,** opq** pl, cpx, ap	hbl 61 opq 13	< 0.5–5.5	28
*3*	*Monzodiorite*,* Monzonite*	2	**kfs**,** pl**, cpx, hbl, opq ± ap ± bt	kfs, 7.8–23.2 **(15.7)** pl, 60.9–78.3 **(69.6)**	1–9	59–77 **(67.8)**

Classification and nomenclature of plutonic rocks from Streckeisen (1974). Major mineral phases (> 5 vol%) are in bold and minor mineral phase (< 5 vol%) in normal text, with abbreviations: olivine (ol), clinopyroxene (cpx), hornblende (hbl), plagioclase (pl), k-feldspar (kfs), feldspathoid (foid) *haüyne, oxide (opq), apatite (ap), biotite (bt), rutile (rt). *hornblende-bearing lithologies include, hornblendite, foid hornblendite, pyroxene hornblendite, hornblende pyroxenite and hornblende gabbro.

**Table 3:** Compositional range and average interstitial melt abundance of the juvenile nodules alongside pumice whole-rock and glass MgO ranges from the Tenerife eruptions

Eruption	*n*= juvenile nodule samples	Interstitial melt vol (range)	Average interstitial melt vol%	Interstitial melt MgO (range)	*n*=	Pumice/glass MgO† (range)	*n*=
Caleta	37	0.6–53%	20.3%	1.3–5.5 wt%	12	0.1–2.9 wt%	47
Fasnia	103*	5.0–63%*	26.4%*	1.1–9.2 wt%*	56	0.1–2.8 wt%	239
San Juan	9	5.6–33.7%	19.4%	2.8–5.2 wt%	4	0.7–1.6 wt%	6
Morteros	20	1.4–62.6%	20.4%	4.1–5.5 wt%	7	0.6–1.0 wt%	2
Gaviotas	20	0.8–77.0%	22.4%	4.8–7.1 wt%	7	0.9 wt%	2
***Tenerife interstitial melt modal average***			***24.6%***				

Only includes melt-bearing samples and based on point counts (full dataset in Supplementary Material S2). *Fasnia data available in Horn et al. (2022). †Whole-rock pumice data and glass data from this study, Olin 2007; Dávila-Harris et al. 2023; Horn et al. 2022). MgO is used over SiO_2_ to allow comparison between XD-WRF and ICPMS data. Analytical method for each given data in this study is given alongside the measurement the Supplementary Material S2.

**Table 4:** Summary of the average major element glass compositions from the Caleta and Fasnia units from this study and bulk-rock pumice data (*) from Dávila-Harris et al. (2023) for the San Juan, Morteros and Gaviotas eruptions

Eruption/sub-units	*n*=	SiO_2_ (wt%)	TiO_2_ (wt%)	Al_2_O_3_ (wt%)	FeOt (wt%)	MnO (wt%)	MgO (wt%)	CaO (wt%)	Na_2_O (wt%)	K_2_O (wt%)	*P*_2_O_5_ (wt%)	Cl (wt%)
*Caleta*	46	60.35	0.59	20.72	2.28	0.15	0.29	1.51	8.24	5.54	0.07	0.26
*Fasnia all units average*	135	57.96	0.44	21.69	2.53	0.19	0.24	0.97	9.78	5.73	0.06	0.40
*Fasnia Atogo ignimbrite*	22	58.70	0.40	21.71	2.39	0.20	0.22	0.96	9.29	5.72	0.05	0.37
*Fasnia Unit M*	23	57.79	0.44	21.55	2.41	0.20	0.20	0.83	10.33	5.80	0.06	0.38
*Fasnia Unit L*	17	58.76	0.53	21.37	2.48	0.17	0.25	0.94	9.10	6.00	0.04	0.37
*Fasnia Unit H*	4	59.19	0.39	22.52	2.41	0.20	0.16	0.81	8.00	5.83	0.03	0.45
*Fasnia Upper units average*	*66*	*58.43*	*0.45*	*21.62*	*2.42*	*0.19*	*0.22*	*0.90*	*9.53*	*5.83*	*0.05*	*0.38*
*Fasnia Ravelo*	26	57.73	0.37	21.74	2.42	0.19	0.18	0.79	10.22	5.87	0.04	0.46
*Fasnia Maracay ignimbrite Unit C*	29	57.34	0.56	21.60	2.87	0.20	0.40	1.37	9.71	5.47	0.11	0.37
*Fasnia Unit A-C*	14	57.45	0.34	22.13	2.51	0.21	0.18	0.80	10.33	5.58	0.04	0.43
*Fasnia Lower units average*	*69*	*57.51*	*0.44*	*21.76*	*2.63*	*0.20*	*0.27*	*1.04*	*10.03*	*5.65*	*0.07*	*0.41*
San Juan*	6	61.53	0.94	16.43	4.17	0.27	1.05	1.23	8.82	5.45	0.13	
Morteros*	2	60.04	0.86	19.23	3.03	0.16	0.77	1.61	8.66	5.50	0.14	
Gaviotas*	2	60.88	1.01	18.71	3.50	0.18	0.95	1.70	8.12	4.74	0.22	

Averages represent normalised analytical totals; a complete dataset is provided in the Supplementary Material S2.

Details of the Fasnia eruption sequence are detailed in Edgar et al. (2017).

Regional correlation of the older Las Cañadas deposits is more fragmented due to variable degrees of outcrop exposure and erosion but with detailed mapping provided in Dávila-Harris (2009; 2023), Martí et al. (1994), and Soriano et al. (2006). Formations in the Upper Group, listed in order of age, include the Ucanca (1.66–1.07 Ma: Martí et al. 1994; Ancochea et al. 1999), Guajara (0.85–0.57 Ma: Martí et al. 1994, 1997), and Diego Hernández Formation (DHF; 0.37–0.17 Ma: Martí et al. 1994, 1997; Edgar et al. 2007; Brown et al. 2003).

The Diego Hernández Formation (DHF; Fig. 1c) is the most extensively studied volcanic sequence on Tenerife, providing a well-characterised but complex stratigraphic and geochemical framework (Walker 1981; Wolff 1985; Martí et al. 1994; Bryan et al. 1998, 2002; Martí and Gudmundsson 2000; Edgar et al. 2007; Cas et al. 2022). Its detailed study is crucial for understanding magma mixing as a trigger for eruptions, evidenced by reversely and normally zoned phenocrysts, quenched mafic glass blebs in pumice, banded pumice, and bimodal phenocryst and glass compositions (Wolff 1985; Olin 2007; Wolff et al. 2020; González-García et al. 2022). These well-documented stratigraphic relationships, geochronological constraints, and petrographic and geochemical signatures make the DHF an ideal reference for contextualising new samples. Work by Horn et al. (2022) showed that the Fasnia eruption ejected melt-bearing, partially crystalline cumulate nodules that preserved the crystal framework and pre-eruptive melt within an active magma mush reservoir. Those results indicated that the mafic-felsic mush and phonolite existed as separate crustal reservoirs with minimal two-way mixing, suggesting a layered magmatic system with the mafic reservoir underlying the felsic one.

Tenerife’s magmatic system is geochemically complex, and systematic chemical variations preserved in the pyroclastic stratigraphy show that the controlling processes operated on timescales of 100–1000 ka (Dávila-Harris et al. 2023). Geochemical variations in phonolites result from a combination of fractional crystallisation (Neumann et al. 1999) and melting of crystal-rich mushes (Sliwinski et al. 2015), particularly feldspar-rich cumulates, as evidenced by positive Eu anomalies (Wolff et al. 2015, 2020). These processes are influenced by changes in the pressure and temperature of the phonolite reservoirs, which in turn control the relative proportions of crystallising phases such as alkali feldspar, plagioclase, clinopyroxene, amphibole, and biotite. Major element variations in Tenerife magmas primarily reflect fractional crystallisation, whereas trace element profiles are additionally influenced by syenite and basement rock assimilation (Wolff and Palacz 1989; Ablay et al. 1998; Wolff et al. 2000), and interactions at different crustal levels further modify these signatures (Turner et al. 2017). The relationship between the shallow, melt-rich phonolite reservoir and crystal cumulate signatures is well studied (Pittari et al. 2008; Sliwinski et al. 2015; Wolff et al. 2015, 2020; Dorado et al. 2023). For instance, Nb-Zr relations help define chemostratigraphic divisions and phonolite groups (Dávila-Harris et al. 2023), with varying Nb enrichment indicating different roles for residual titanite during phonolite petrogenesis, either through fractional crystallisation or the melting of pre-existing syenites (Wolff et al. 2000; Edgar et al. 2007; Cas et al. 2022). It has been hypothesised that crystal-poor magmas are extracted from these mush reservoirs (Sliwinski et al. 2015). Phase equilibrium experiments estimate the phonolites at the top of the reservoir evolved within a magma chamber approximately 4–5 km (825 ± 25 °C, 1.3 ± 0.5 kbar) below the surface (Andújar et al. 2008; Martí et al. 2020; González-García et al. 2022). However, the crustal magma reservoir architecture and the degree of interaction between the phonolite reservoir and the mafic mush remain poorly constrained within the magma plumbing system. Unlocking the interface between the melt-rich regions and mush is central to understanding the chemical evolution of the crustal magma reservoir and the behaviour of volcanic eruptions on Tenerife.

## Methods

### Samples and stratigraphy

We use the term juvenile nodules to refer to the plutonic clasts containing a juvenile microcrystalline groundmass component. Following Cooper et al. (2016), we avoid defining the samples as “cumulates”, due to the inferred interpretation of emplacement mechanism and bulk sample composition. The juvenile component separates them from holocrystalline, and often altered, plutonic samples that form an occasional part of the lithic clast assemblage within Tenerife ignimbrite breccias (Pittari et al. 2008). Based on their crystal and groundmass textures and their particle volume the juvenile nodules are characterised as ‘mush’ (Cashman et al. 2017). The microcrystalline groundmass provides key evidence that the samples were supra-solidus at the time of the eruption and part of the active crystal-rich mush reservoir (Stock et al. 2012; Cooper et al. 2016; Chamberlain et al. 2019; Horn et al. 2022). Here, we refer to the groundmass component as interstitial melt, abbreviated to ‘melt’ when relating to the geochemistry of the groundmass.

The stratigraphic nomenclature for Tenerife is complex, varying between studies (summarised in Cas et al. 2022). Here, we use the stratigraphic hierarchy from Martí et al. (1994) and Dávila-Harris (2009; 2023), referring to each eruption by its most recently defined name as shorthand. We have sampled nodules from lithic-rich horizons across multiple pyroclastic deposits within the Tenerife volcanic stratigraphy (Fig. 1c), including the Caleta and Fasnia eruptions of the DHF, the San Juan eruption (Ucanca), and the Morteros and Gaviotas eruptions (Lower Group deposits). Juvenile nodules and lithic clasts are not exclusive to these deposits and have also been sampled from other DHF eruption units such as the Abrigo ignimbrite (e.g., Pittari et al. 2008), and the Poris eruption (Stock et al. 2012).

**Fig. 2:**
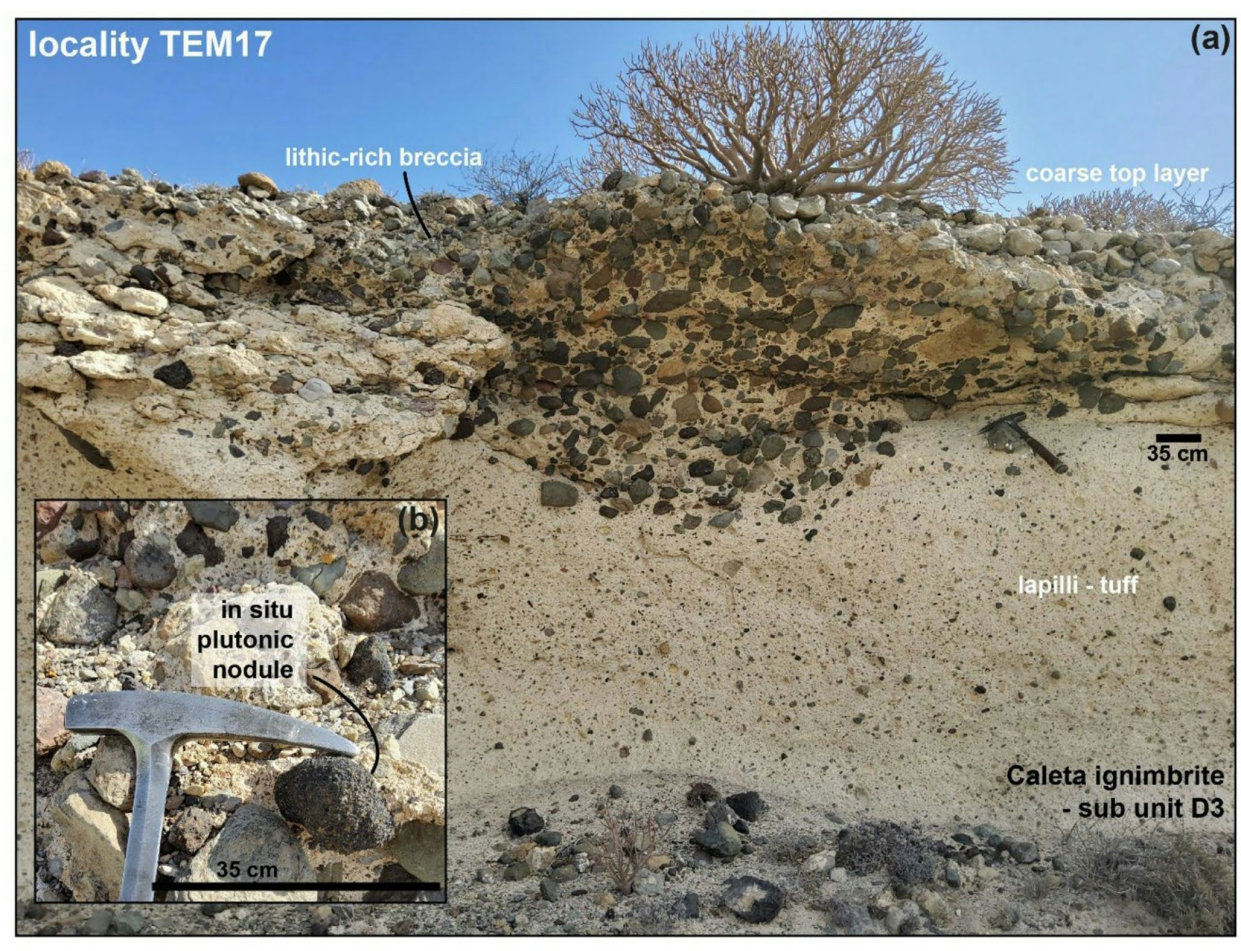
Field photos from the lithic breccia facies of sub-unit D, Caleta ignimbrite. **a** Section of the deposit at locality TEM-17, sample location grid references are in the Supplementary Material. **b** Insert, photo of in situ nodule, sample TEM-17E. Photo orientation: facing NE.

The ages and key characteristics of these eruptive units are summarised in Table 1. The Caleta eruption has an ^40^Ar/^39^Ar age of 221 ± 5 ka (Brown et al. 2003), with the deposit characteristics described in detail in Bryan et al. (1998), Brown et al. (2003) and Edgar et al. (2007). The Caleta consists of 5 units, with Unit D comprising the Caleta ignimbrite. Lithic clasts, melt-bearing nodules and other coarsely crystalline nodules are found in the lithic-rich breccia of sub-unit D3; these clasts are block/boulder in size and the lithic breccia horizon occurs towards the top of the ignimbrite (Fig. 2a; Bryan et al. 1998). Caleta juvenile nodules are between 12 and 90 mm in size (Fig. 2b). The Fasnia sequence, dated at 312 ± 6 ka, consists of a complex sequence of units with eight principal ash horizons, seven magmatic Plinian fallout units and seven main ignimbrite units (Edgar et al. 2007, 2017). Fasnia nodule samples are predominantly found in the Ravelo ignimbrite, as detailed in Horn et al. (2022). Detailed descriptions, stratigraphic logs and key outcrop locations of the San Juan, Morteros and Gaviotas are presented by Dávila-Harris (2009; 2023). The Playa de San Juan Ignimbrite, dated at 1.5 ± 0.03 Ma (Huertas et al. 2002; Dávila-Harris et al. 2023), is recognisable for its green, eutaxitic lapilli-tuff at the base and is capped by a lithic-breccia where nodules are found and described as feldspar-rich juvenile blocks by Dávila-Harris (2009). The type locality for the Morteros is at Punta Gaviotas (Dávila-Harris 2009), where it unconformably overlies the Gaviotas. The Morteros age is estimated between 1.6 and 1.8 Ma (Taylor et al. 2020), as a stratigraphic estimate between the underlying Gaviotas at 1.840 ± 0.070 Ma (Huertas et al. 2002) and overlying Enramada at 1.662 ± 0.02 Ma (Dávila-Harris et al. 2023). The Gaviotas ignimbrite is distinguishable by its orange/brown colour and a diffuse-bedded lithic breccia (Dávila-Harris 2009). Again, nodules were sampled from the thick lithic-rich breccia facies, with very few samples found in the finer lapilli-tuff facies of the units. We also present new glass data from samples collected from the Fasnia and Caleta, from both the DHF caldera wall exposures and coastal deposits; detailed sampling locations are provided in Horn (2023). Pumice and ash were sampled from Caleta fall and ignimbrite units, while units sampled in the Fasnia Lower sequence include Units A-C, Maracay ignimbrite, and Ravelo ignimbrite, and in the Fasnia Upper sequence include Units H, L, M, and Atogo ignimbrite (cf. Edgar et al. 2017 for a full description of the Fasnia eruption sequence). Sample geolocations (Fig. 1a) are available in the Supplementary data, and all samples are prefixed with their location name (i.e., L1-X, TEM-X).

### Analytical techniques

Nodule samples were cut, prepared as polished thin sections, imaged as optical photomicrographs and point counted, following the methods described by Horn et al. (2022). In total, mineral modal abundance were point counted for: 46 Caleta nodules, 11 San Juan nodules, 20 Morteros nodules and 20 for the Gaviotas nodules. We integrate these data with petrographic information for a further 111 juvenile and xenolith samples from the Fasnia, published by Horn et al. (2022).

Mineral analysis, backscattered electron images and major element analyses of olivine, clinopyroxene, plagioclase, K-feldspar, feldspathoid, amphibole, biotite, zircon, oxide, and apatite crystals were collected using a Zeiss 1450VP scanning electron microscope (SEM) in the School of Ocean and Earth Science, University of Southampton, equipped with an Oxford Instruments silicon drift energy dispersive spectrometer (EDS). SEM analyses of all phases and combined Backscatter Electron Imaging (BSE) and elemental maps are presented in the Supplementary Material. EDS major element data were collected with 20 kV accelerating voltage and the instrument was calibrated using Oxford Instruments factory standards.

Elemental analyses from clinopyroxene, olivine, amphibole and plagioclase crystals from nodule samples were also measured using a wavelength dispersive (WDS) Cameca SX100 electron microprobe (EMP) in the School of Earth Sciences, University of Bristol, U.K. Analyses were focussed on these phases as they were dominant across the sample collection and provide the most direct constraints on crystallisation conditions and differentiation processes, also ensuring comparability with previous work. Mineral analyses were collected using a 20 kV, 20 nA, focussed (∼1 μm) beam, with peak count times 10–30 s for major elements (> 1 wt%) and 30–80 s for minor elements (< 1 wt%). Analyses with totals outside 96–102 wt% were excluded. Analytical uncertainty was examined through repeat analyses and comparison of Smithsonian Microbeam Standards (Jarosewich et al. 1980), relative precision (2σ) is better than ~ 1–2% for major elements, except Na_2_O (± 2.9%) and FeO (± 4.1%), and better than ~ 3–5% for minor elements, except Cr_2_O_3_ (± 10.8%) and MnO (± 34.4%). Pyroxene formula recalculations are on a six-oxygen (6O) basis and phase components are defined according to Putirka (2008). Major and minor elements have been recalculated as atoms per formula unit (a.p.f.u) using the MINERAL software (De Angelis and Neill 2012). We also include results from Tenerife nodules presented in the thesis of Bromhead (2013); where olivine, clinopyroxene, amphibole, magnetite and plagioclase were measured using a JEOL8600 wavelength dispersive electron microprobe (WDS-EMP) at the Research laboratory for Archaeology and the History of Art (RLAHA), University of Oxford, U.K. A full description of the analytical conditions and standards are given in Bromhead (2013) and results are also included in the Supplementary Material.

We present new glass major element analyses of 12 samples from the Fasnia and Caleta eruption units. Glass analyses were restricted to these two eruptions as fresh pumice was available from these units. In other eruptions, pumice was deemed to be too altered to yield reliable in-situ glass analyses, and bulk-rock analyses would not have provided a comparable record of melt composition. Samples for glass chemistry were prepared and analysed at RLAHA, University of Oxford, U.K. Bulk samples comprising pumice and ash were crushed and wet sieved at 80 μm, dried and mounted in epoxy. Major elements were analysed using a JEOL JXA-8200 wavelength dispersive electron microprobe (WDS-EMP) with an accelerating voltage of 15 kV, a 6 nA beam current and a beam diameter of 10 μm. Peak counting times were 12 s for Na, and other major elements collected for 30 s except for Mn, Cl and P which were collected for 50 s. Analyses with totals outside 94–102 wt% were excluded and values from secondary standard reference glasses (ATHO-G, StHs6/80-G and GOR128-G; Jochum et al. (2005); http://georem.mpch-mainz.gwdg.de) for each analytical session are presented in the Supplementary Material. Based on repeat analysis of the StHs6/80-G standard, relative errors (RSD) for: SiO_2_ ±0.8%, TiO_2_ ±7.9%, Al_2_O_3_ ±1.7, FeOt ± 4.8%, MgO ± 3.8%, CaO ± 2.2%, Na_2_O ± 7.3%, K_2_O ± 4.3%, MnO ± 44% and P_2_O_5_ ±66%.

Trace element composition of the nodule microcrystalline groundmass was measured following the methods in Horn et al. (2022). Major and trace element analyses were made on 27 interstitial melt samples using a Thermo Scientific X-Series II inductively coupled plasma-mass spectrometer (ICP-MS) in the School of Ocean and Earth Science, University of Southampton. Data were corrected for interferences and an analytical blank, prior to calibration using a suite of international rock standards (JB-3, JB-1a, JGb-1, BHVO-2, BIR-1, JA-2; Jochum et al. 2005) and in-house reference material BRR-1. 2σ analytical precision was determined from repeat analyses of international secondary standard JA-2 and is < 0.1% for rare-earth elements (REE), except Ce (1.3%) and Nd (0.5%). For other elements of interest in this study 2σ is 1.9% for Zr and 1.2% for Nb.

Combined modal mineral abundance data, SEM, EMPA and ICP-MS datasets for melts and minerals, including backscattered electron images (BSE), elemental maps and the position of SEM analysis sites along with an assessment of analytical accuracy are provided in the Supplementary Material (Fig. S5).

**Fig. 3:**
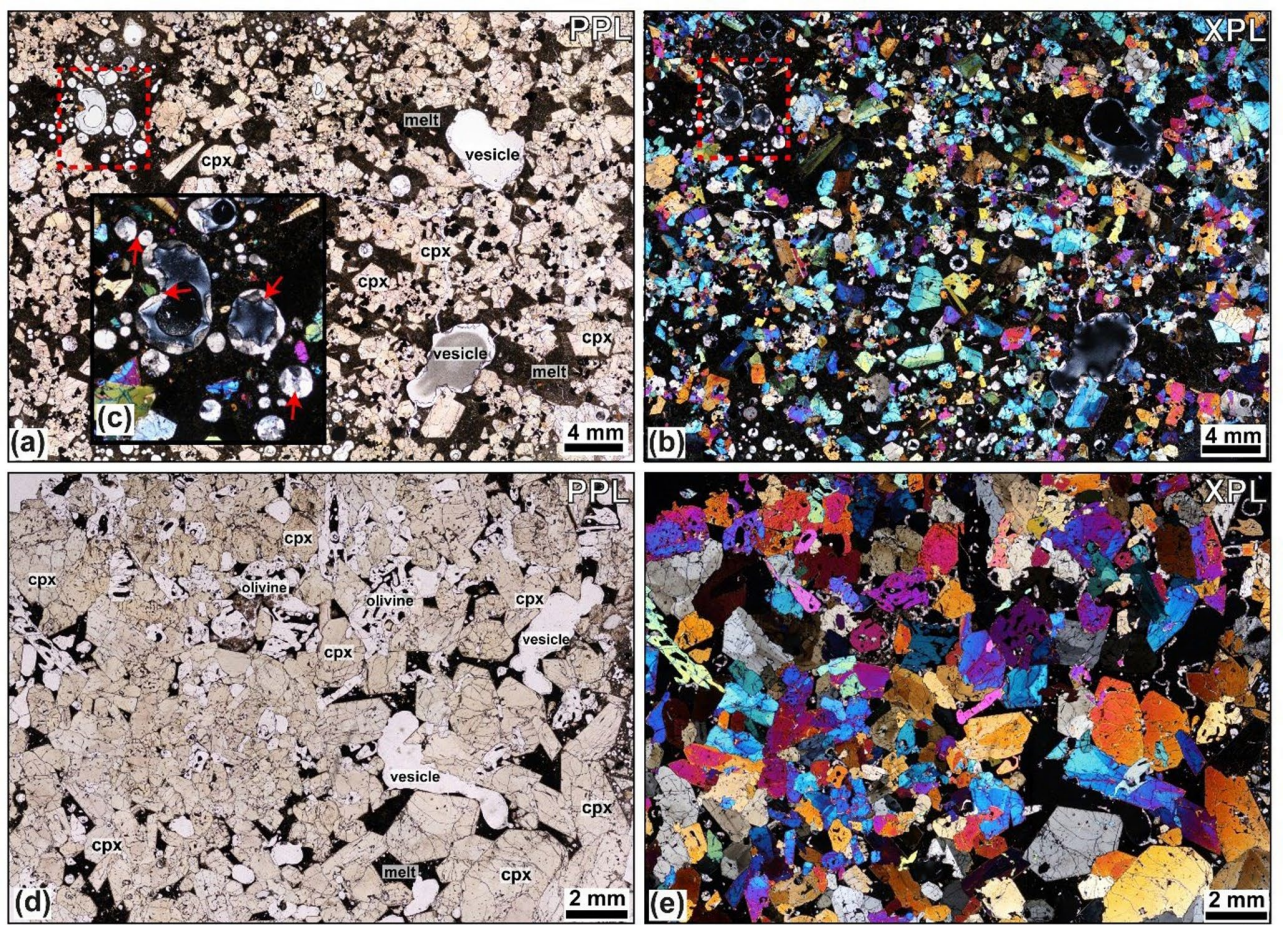
Caleta ultramafic nodules. **a**–**c** Sample L2-16 clinopyroxenite with calcite filled vesicles, marked by red arrows in insert **c**. **d**–**e** Sample TR018-04 wehrlite sample, with examples of subhedral embayed olivine and sector zoned clinopyroxene.

## Results

### Juvenile nodules textures

**Fig. 4:**
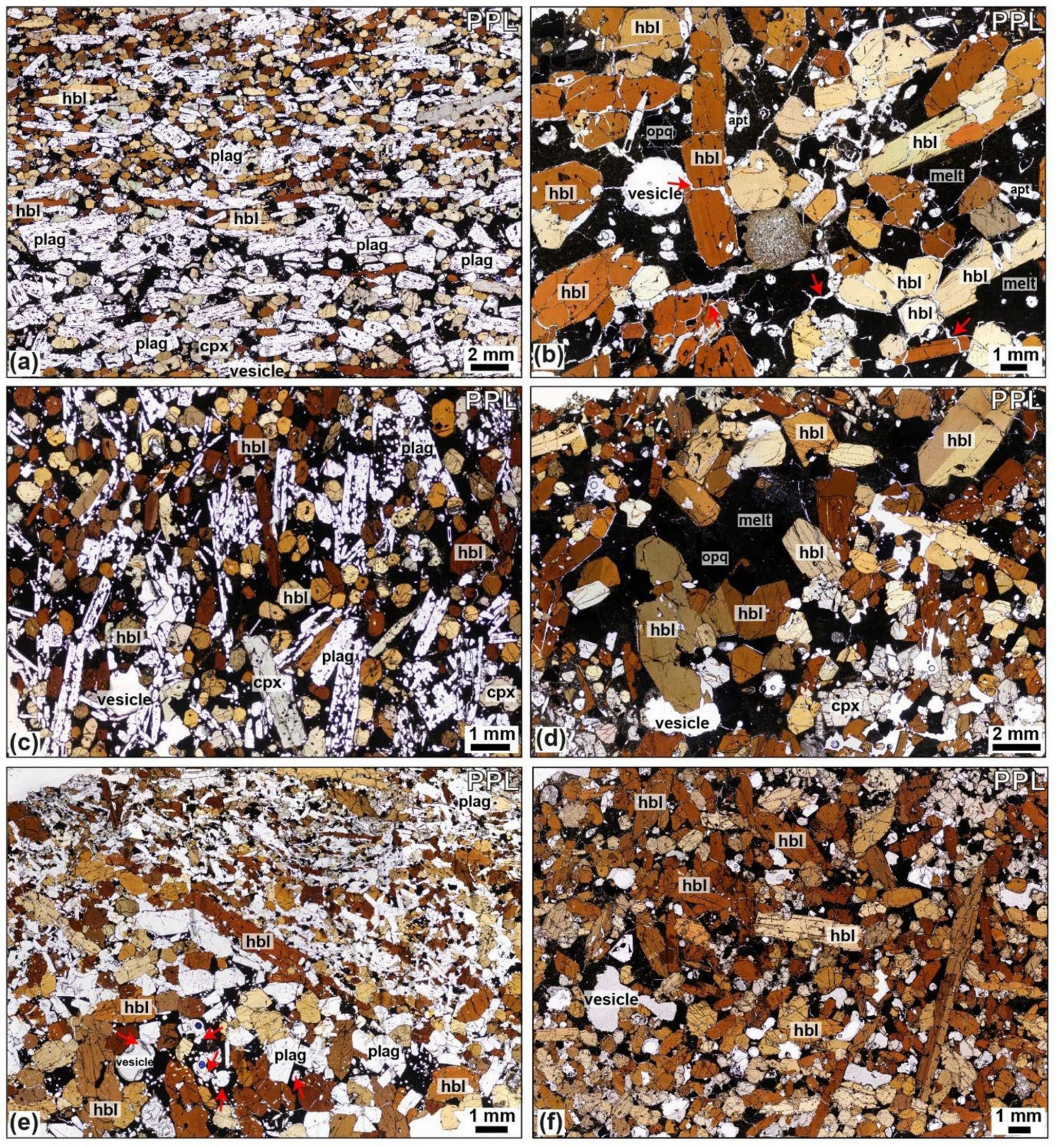
Hornblende-gabbro Caleta nodules, showing different environments of crystallisation through lineation of tabular minerals and layering. Samples are as follows: **a** L2-21. **b** L2-94. **c** L2-49. **d** L2-18. **e** L2-28. **f** L2-12. Red arrows highlight examples of cracks in crystals in **b** and partially calcite-filled vesicles in **e.**

**Fig. 5:**
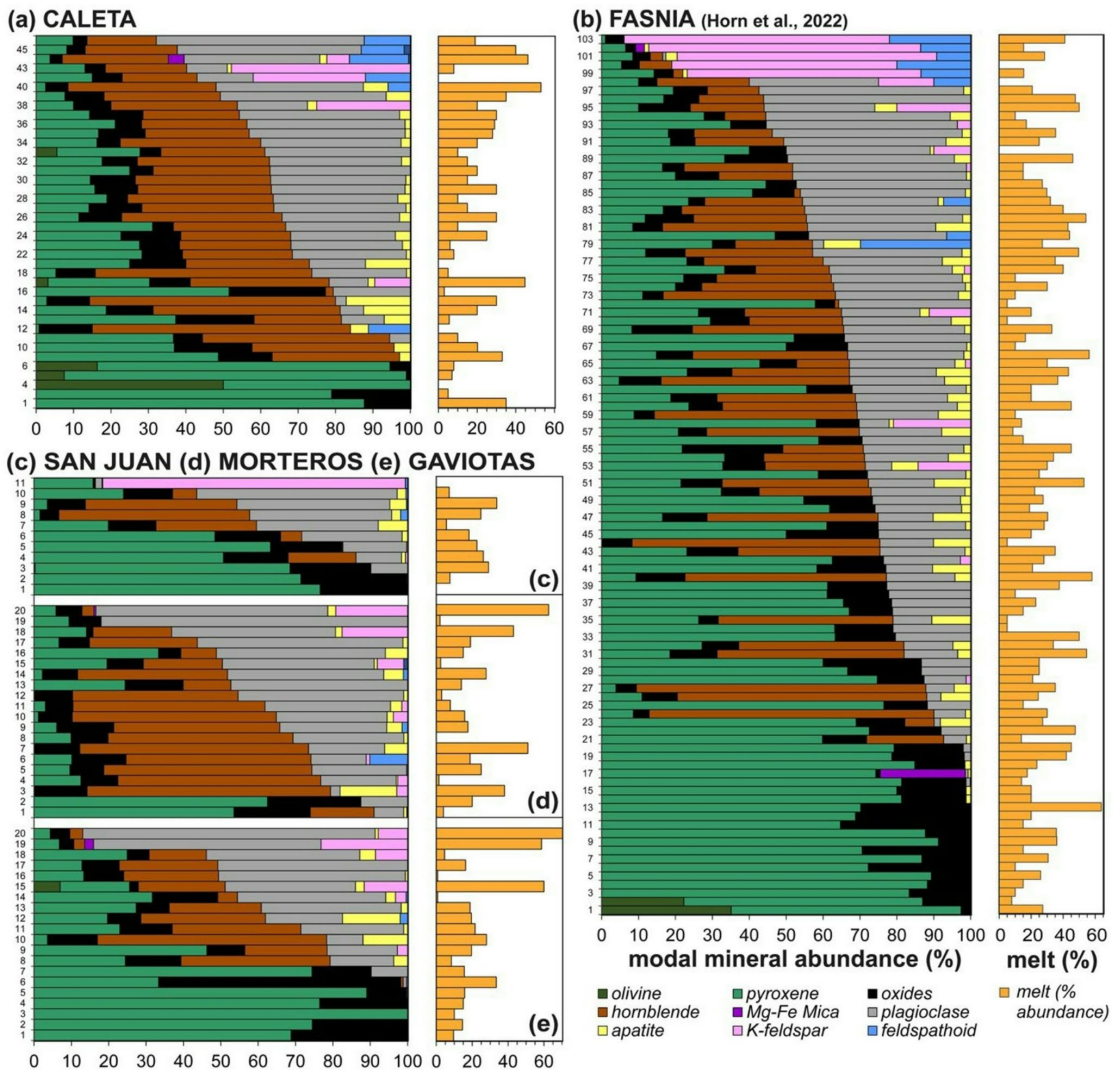
Petrology summary and mineral abundances for the Tenerife nodule collection, sample numbers are marked on the left-hand side and correspond to the modal abundance data for the Caleta, San Juan, Morteros and Gaviotas (given in the Supplementary Material S1). Three distinct nodule groups based on lithology classification (1) ultramafic nodules, (2) gabbroic nodules, (3) felsic nodules, (described in text and in Horn et al. 2022). **a**: Caleta **b** Fasnia (modified after Horn et al. 2022). **c**: San Juan **d**: Gaviotas **e** Morteros. Left panels shows normalized modal mineralogies ordered with increasing proportion of mafic minerals down the stacked bar chart. Right panels give the estimated proportion of interstitial melt from each sample.

The juvenile nodules predominantly consist of coarse crystals and are classified using the scheme of Streckeisen (1976). They range from porphyritic to equigranular, showing variably developed textures, from orthocumulate to adcumulate textures across the sample set. Crystal sizes range from < 0.5 to 18 mm, and textures include aplitic, anhedral to subhedral, seriate, and hypidiomorphic-granular. Most nodules are phaneritic and exhibit equilibrium textures, though reactive and disequilibrium features such as sieve-textured feldspars are common across all lithological types, bar the ultramafic lithologies (Fig. 3), as identified in Fasnia nodules (Horn et al. 2022). Plagioclase is tabular and euhedral to subhedral. Sieve-textured plagioclase, featuring fragmented crystals or groundmass-filled cores, is common (Figs. 4c and 5a), and other samples show equilibrium plagioclase (Fig. 5b, d). Clinopyroxene shows multiple zoning textures, including oscillatory, sector, and concentric zoning, with well-defined cores and rims. Narrow, well-defined rim zones are visible in thin section where minerals contact with the groundmass (Stock et al. 2012; Horn et al. 2022). Crystal boundaries vary from non-planar and irregular, amorphous when in disequilibrium, to planar, with minerals often displaying perfect crystallographic shapes. Glomerophyric and glomeroporphyritic textures are present in some samples, with poikilitic textures observed.

**Fig. 6:**
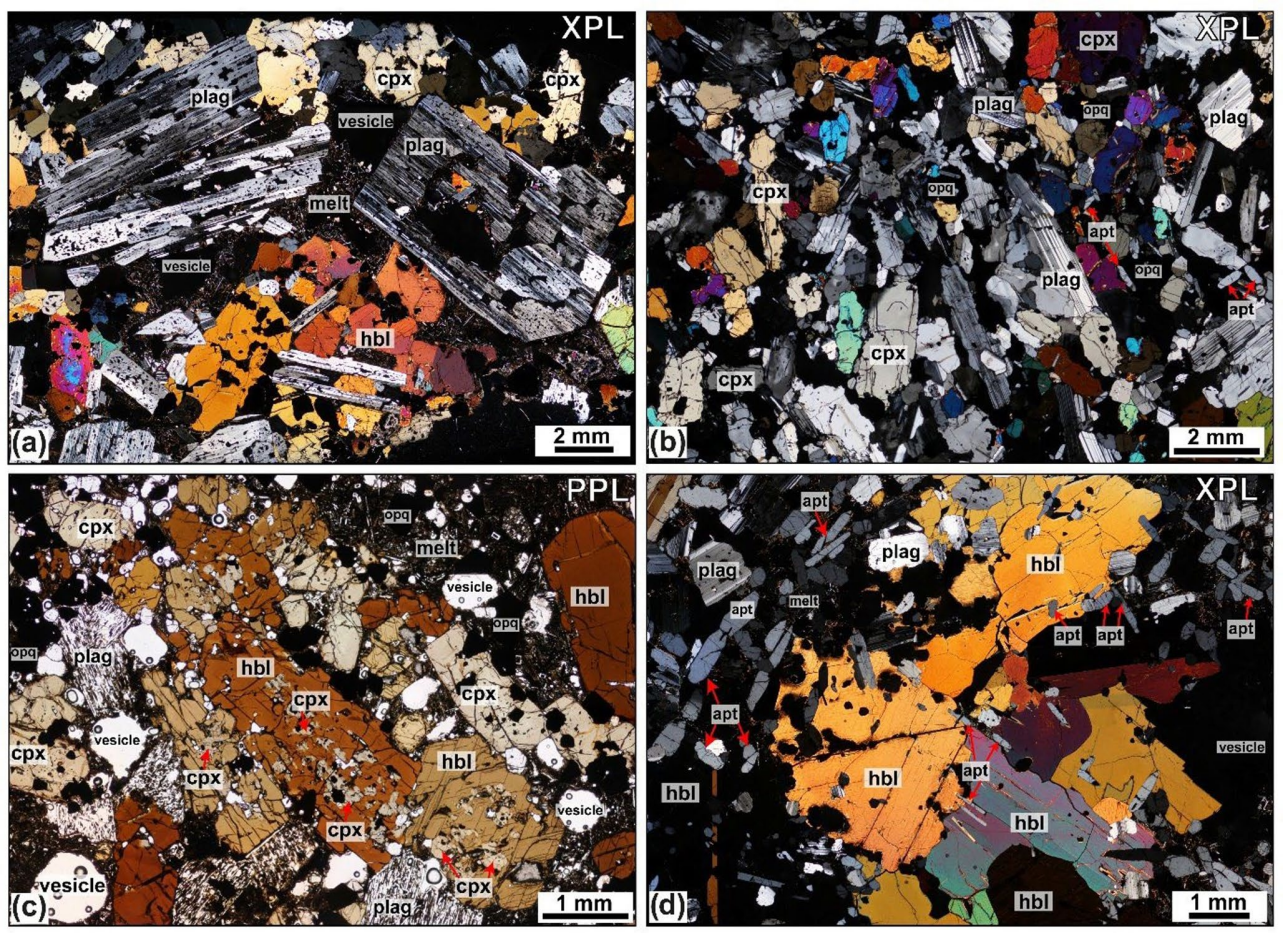
Gaviotas hornblende-gabbro nodules **a** Sieve textured plagioclase, dissolved core and mantle of plagioclase in XPL from sample TR028-08. **b** Plagioclase in equilibrium from TR027-03. **c** Poikilitic texture with clinopyroxene mantled by hornblende from sample TR028-13. (d) Elongate euhedral apatite inclusions in hornblende from sample TR027-01, apatite examples labelled with red arrows.

The crystallisation environments of these textures can be divided into in-situ growth and crystal accumulation, as identified previously in the Fasnia nodules (Horn et al. 2022). Samples showing strong crystal alignment, indicating movement of crystals relative to the groundmass, are interpreted to have formed via crystal deposition in a liquid-rich magma body (Wiebe and Collins 1998; Holness et al. 2019). Crystals are moderately equigranular, with plagioclase and hornblende showing the strongest lineation (e.g., Fig. 4a, c, e). Layering is common within these nodules, defined by variations in grain size, grain shape, orientation, and mineral modal abundance, with multiple layering types found within the same sample. In contrast, samples where cumulus crystals impinge on one another, or where the groundmass fills the pore space within interstitial regions created by the grain framework, are interpreted as having formed in-situ within a static growth environment (Wager & Brown 1967; Holness 2014; Cooper et al. 2016; Holness et al. 2019; e.g., Fig. 4d). The key petrographic features of the Tenerife juvenile nodules are summarised in Table 2, detailing their lithology, mineral abundances, and grain size range in each eruptive unit.

### Juvenile nodule petrography

The plutonic nodule samples comprise wehrlite, clinopyroxenite, pyroxene hornblendite, pyroxene hornblende gabbro, gabbro, feldspathoid syenite, monzodiorite or monzonite. Samples are arranged in a stack (Fig. 6) with mafic mineral content (i.e., the proportion of olivine, clinopyroxene, amphibole and oxides relative to feldspar, feldspathoid and apatite) increasing top-to-bottom. From petrological analysis of Fasnia eruption, three groups of nodules were identified based on key changes in modal mineralogy (Horn et al. 2022): ultramafic nodules, comprising clinopyroxenites and wehrlites, which are plagioclase-free; gabbroic nodules, including pyroxene hornblende gabbros, hornblende gabbros, and gabbros; and felsic nodules, characterised by over 80% feldspars and feldspathoids, categorized as feldspathoid syenites and monzodiorites. Nodule samples from the Caleta (*n* = 44), San Juan (*n* = 11), Morteros (*n* = 20), and Gaviotas (*n* = 20) eruptions have been point counted, compared to the Fasnia samples (*n* = 103); same mineralogical groupings can be identified (Table 2). See Horn et al. (2022) for full detail of the Fasnia nodule samples.

### Ultramafic nodules

In four of the five units sampled; a sub-set of nodules contain only mafic minerals (inclusive of the Fasnia nodule data). These ultramafic wehrlites and clinopyroxenites (Fig. 3) form 11%, 18% and 30% of the Caleta, San Juan and Gaviotas nodule suites, respectively (Table 2). No ultramafic nodules were found in the Morteros suite. In these samples, clinopyroxene crystals reach 0.6–6.8 mm, olivine crystals range 0.5–7.2 mm, and oxides are 0.1–6 mm, often as inclusions within other phases. The modal abundances of minerals in the ultramafic nodules across the eruptions average between 52.0 and 74.0 vol% clinopyroxene, 19.3–23.0 vol% olivine, and 2.7–26.3 vol% oxides (Table 2). The groundmass is glassy with no visible microlites (< 0.1 mm), calcite crystals partially fill some vesicles in the Caleta samples (Fig. 3c), and vesicle morphology is spherical to non-spherical (Fig. 3d-e).

### Gabbroic nodules

**Fig. 7:**
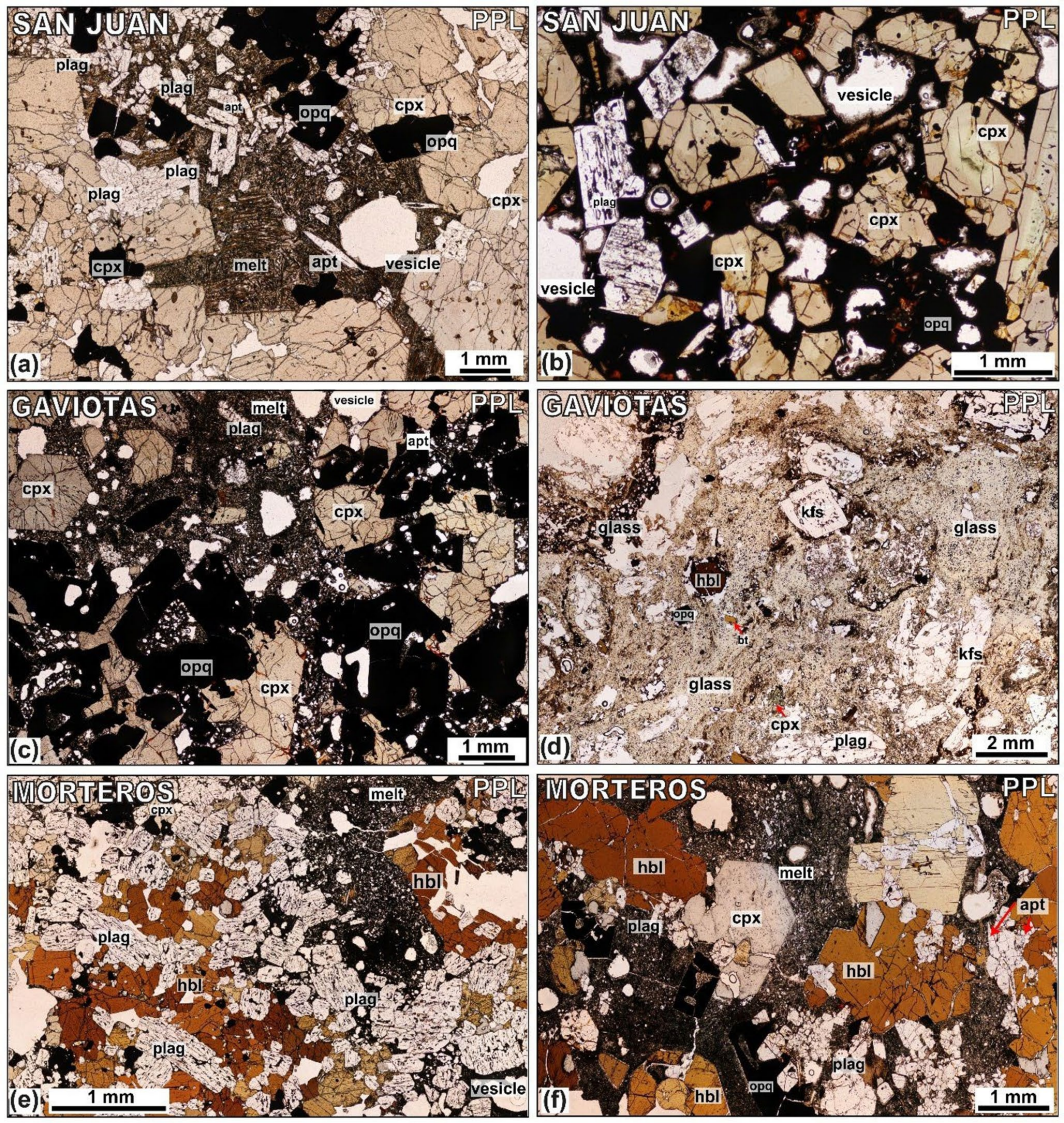
Different interstitial melt textures from San Juan, Gaviotas and Morteros nodules. **a**: Microlite groundmass (melt) from TR022-10 (San Juan). **b**: Dark, glassier groundmass (melt) surrounding clinopyroxene (cpx) and oxides (opq) crystals from sample TR022-08 (San Juan). **c**: Microlite-rich from sample TR027-05 (Gaviotas). **d**: Thin section through a groundmass-rich region and disaggregated cumulus crystals, mixed interstitial groundmass glass from sample TR027-08 (Gaviotas). In the top right of the sample, swirling patterns indicates a darker glassy groundmass with swirling, mixing textures. SEM results from Schofield (2021, MSci thesis) show darker melt regions are trachyte and lighter glass plots in the phonolite field. **e**: Isolated pocket of groundmass (melt) in the top center of the sample TR016-14 (Morteros). **f**: Groundmass-rich section, non-linear pathway through the center of sample TR015-08 (Morteros).

The majority of the nodules are gabbroic and include pyroxene-hornblende gabbros, hornblende pyroxenites, pyroxene hornblendites, and hornblende gabbros (Fig. 6). This group constitutes 72% of the Caleta nodule suite, and 54% of the San Juan suite, 55% of the Morteros suite and 50% of the Gaviotas suite (Table 2). They are mineralogically dominated by plagioclase and hornblende, with clinopyroxene generally subordinate. This gabbroic group exhibits significant textural diversity (e.g., Figs. 4, 5 and 7). Plagioclase is typically tabular to acicular, and euhedral to subhedral, with crystals range 0.2–4.5 mm. Sieve-textured crystals, skeletal grains with groundmass-filled cores, are common (Figs. 5a and c and 7b), although some samples also contain compositionally equilibrated plagioclase (Fig. 6d). Hornblende crystals are 0.2–12 mm and euhedral, and clinopyroxene crystals are 0.3–5.2 mm with variable form. Average modal abundances for the gabbroic nodules from each eruption range from 21.0 to 74.0 vol% clinopyroxene, 13.0-31.4 vol% plagioclase and 16.6–51.6 vol% hornblende (Table 2). Oxides, primarily magnetite, comprise 3.8–12.6 vol% of the gabbroic nodules, with crystal sizes ranging < 0.5–6 mm. Minor apatite (< 8.6 vol%) occurs as inclusions in both pyroxene and hornblende with grainsizes 0.1–3 mm (Fig. 5d). Minor feldspathoid (< 8.4 vol%; haüyne) is rare but present in some nodules.

### Felsic nodules

Felsic nodules include feldspathoid syenite, monzodiorite and monzonite; this group contains the least number of nodules (Fig. 6), comprising only 4%, 15% and 10% of the Caleta, Morteros and Gaviotas samples, respectively. No felsic nodules were found in San Juan. These nodules contain 0.4–7 mm K-feldspar and 0.5–9 mm plagioclase and 0.5–2 mm clinopyroxene. The modal abundances of minerals in the felsic nodules across all the eruptions average between 12.3 and 81.0 vol% K-feldspar and 7.0-69.6 vol% plagioclase (Table 2). All felsic nodules contain minor oxides (< 2.6 vol%), along with other minor or accessory phases such as feldspathoid (haüyne), hornblende, apatite, and biotite, with abundances for these phases ranging from 0 to 8 vol%.

### Interstitial melts

The nodules have a characteristic mush texture with interstitial groundmass in pore space between crystals or in discrete melt pathways. The groundmass contains vesicles which range in size from < 0.05 to 6 mm; some are spherical but most have irregular shapes, filling the interstices between the interlocking crystal frameworks. While the interstitial groundmass material can be glassy, it commonly contains variably sized microlites of acicular, elongate amphibole, oxides, apatite, and feldspar (Fig. 7, S1). These textures range from glassy matrices with sparse, small microlites and dendritic patterns to more crystalline matrices with abundant, larger microlites, comparable to those observed in the Fasnia samples.

For the Caleta eruption, the interstitial groundmass volume ranges from 0.6 to 53% with an average of 20.3% (Table 3). Interstitial groundmass contents vary across eruptions: Fasnia ranges from 5.0 to 63% (avg. volume of 26.4%), San Juan from 5.6 to 33.7% (avg. 19.4%), Morteros from 1.4 to 62.6% (avg. 20.4%), and Gaviotas from 0.8 to 77.0% (avg. 22.4%). Overall, across all of the Tenerife juvenile nodules (*n* = 189 samples combined), the interstitial groundmass modal average for Tenerife nodules is 24.6%. (Table 3).

**Fig. 8:**
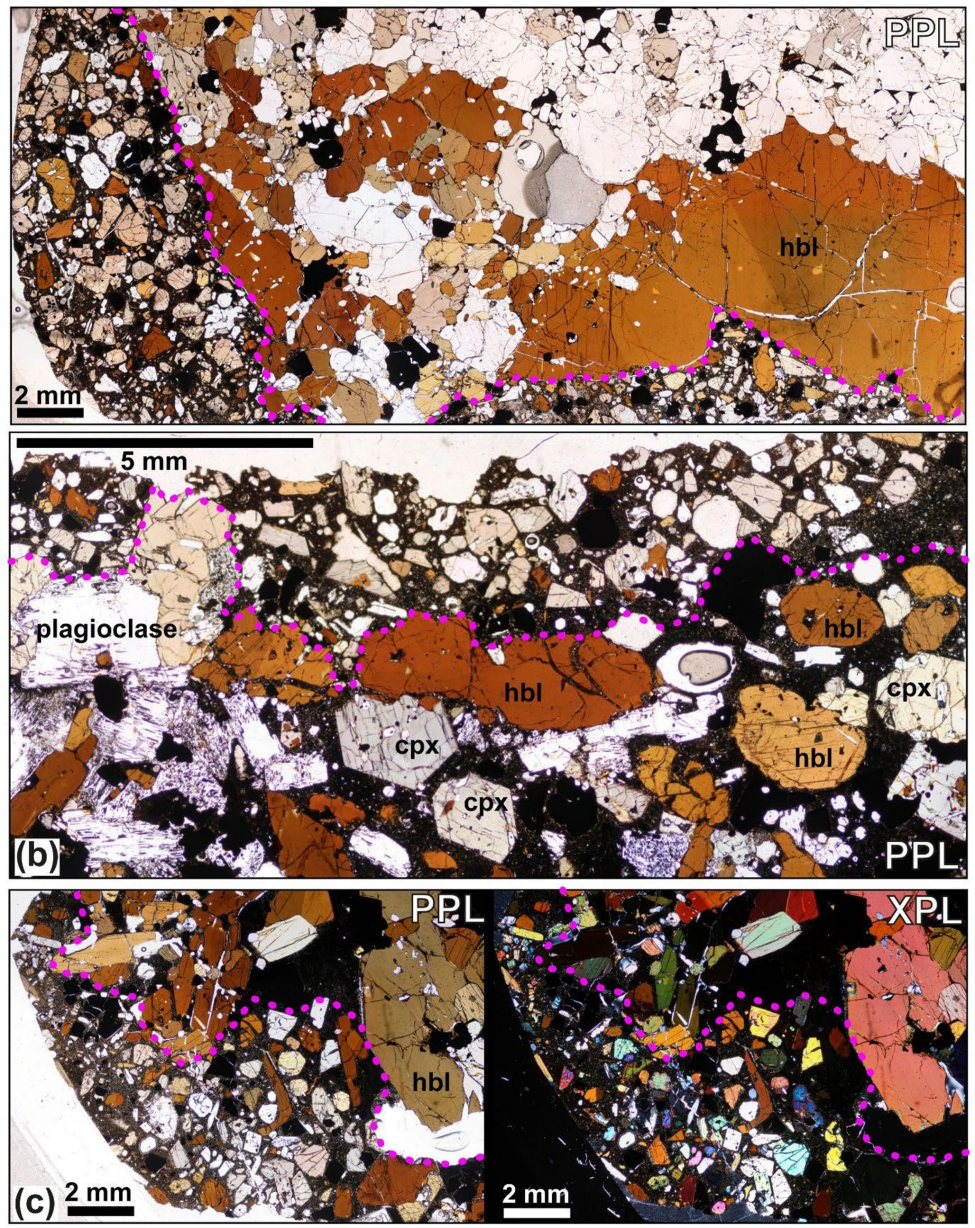
Thin section photographs of samples with groundmass-rich layer in Caleta juvenile nodules, annotated purple line marks boundary of the layer, marking a change in crystal size, textures and groundmass abundance. This layer is found at the edge of the thin sections and hand specimens and contains a mixed crystal assemblage. Samples are as follows; **a** L2-15. **b** L2-15. **c** L2-28.

A sub-group of nine Caleta nodules have a distinct coating (Figs. 8, S2, S3a), observable in hand specimen, which has a different mineralogy and texture to the inner region or core of the nodule sample. This coating is not observed in samples from other eruptions. The coating has a fine-grained microcrystalline to vitrophyric groundmass (< 0.1 mm) with spherical vesicles. Crystals comprise ~  65% of the layer and are typically 1–3 mm, equigranular, subhedral to slightly rounded with very few crystal-crystal contacts. They contain an overall pyroxene-hornblende gabbroic assemblage, but with a diverse mineralogy including olivine, clinopyroxene, hornblende, oxides, plagioclase, with minor apatite and haüyne (Fig. S3c). Contacts between the coating and rest of the sample are irregular and follow the edges of large crystals within the inner nodule (e.g., Fig. S2). Mixing is evident in some Caleta and Gaviotas samples, whereby the coating layer and interstitial groundmass bleed into one another, this is identifiable by their different optical colours (Figs. 7d, S1c).

### Miscellaneous samples

Several Caleta samples are fractured or veined, holocrystalline, and contain abundant biotite; all lack a microcrystalline or glassy groundmass (e.g., Figs. S2, S5a-d). These characteristics match the plutonic lithic clasts described in the Fasnia (Horn et al. 2022) and in the Abrigo ignimbrite (Pittari et al. 2008), where angularity, nepheline feldspathoid assemblages, absence of interstitial groundmass, and strong hydrothermal alteration indicate derivation from older intrusive bodies rather than the active mush. We therefore classify these Caleta samples as xenolithic clasts rather than juvenile nodules. However, some xenoliths are coated by a thin, groundmass-bearing layer (Figs. 8a; S2; S3), also entrained together with the juvenile nodules.

### Phase compositions

#### Mineral chemistry

**Fig. 9:**
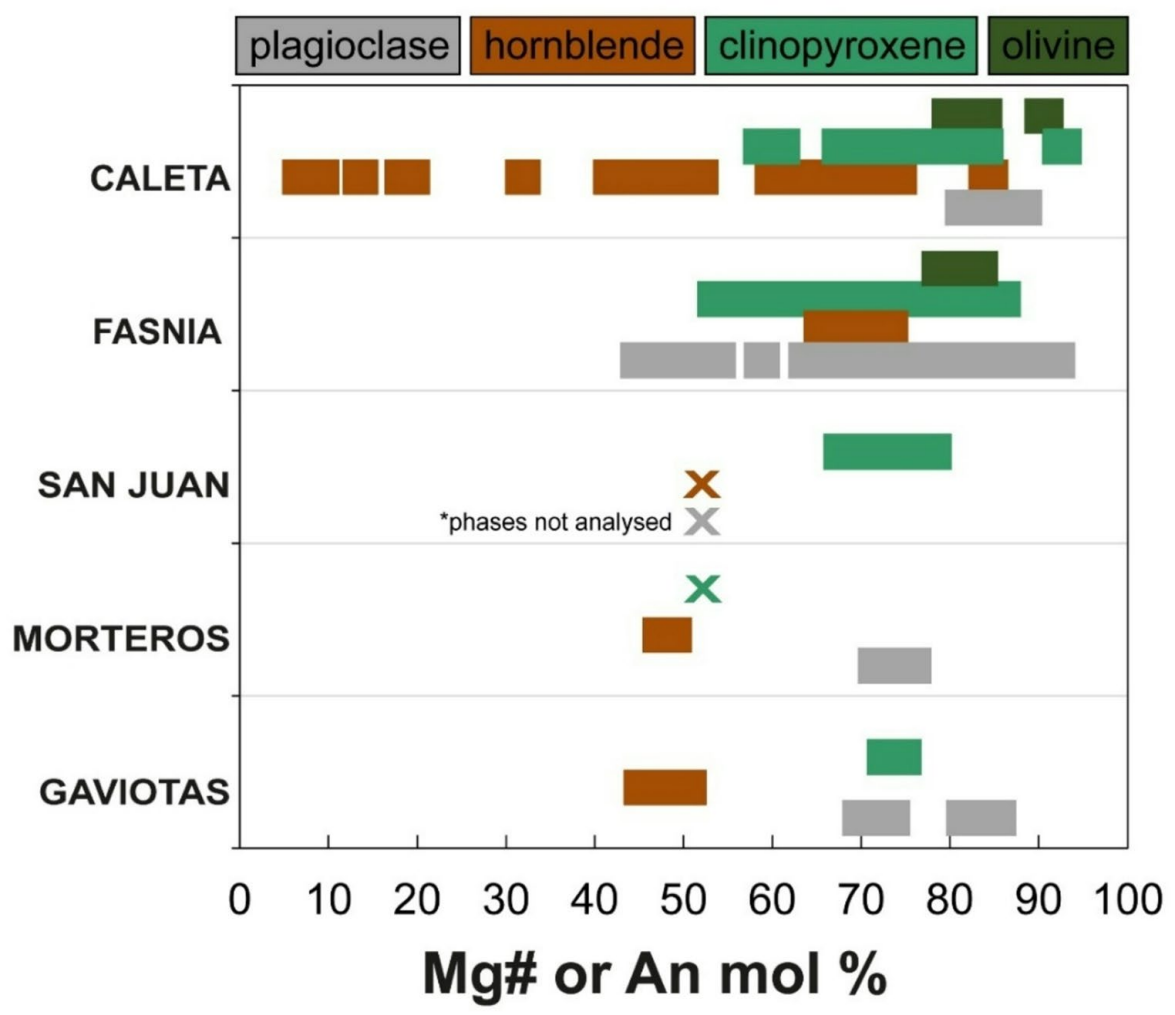
Bar chart summary of phase compositions for all analysed nodules; the range in Mg number (Mg#: expressed as atomic % Mg/[Mg + Fe2+]) of olivine, clinopyroxene and amphibole, and the anorthite content (An = Ca/(Ca + Na + K) of plagioclase. Data from this study, (Fasnia) Horn et al. (2022), and Bromhead (2013). X’s indicate phases that were not analysed.

Mineralogical data for the nodules are summarised through ternary clinopyroxene endmember plots for Caleta, Fasnia, and San Juan (Fig. S6), along with feldspar classification and chemistry, where available (Fig. S8). Comprehensive major element data for the nodules from Caleta, San Juan, Morteros, and Gaviotas are provided in the Supplementary Information (S1). Mineral compositions vary significantly within and between nodules from different eruptions (Fig. 9). In Caleta samples, olivine has Mg# (Mg# = atomic Mg/[Mg+Fe_total_]) ranging from 79 to 90. Hornblende shows a broader variation in Mg#, from 7 to 84. Clinopyroxenes are predominantly diopside (Fig. S6), though some Caleta clinopyroxene exhibits more augitic compositions with high Ti concentrations (> 2.5% TiO_2_; Fig. S7), classifying them as titanaugite. Feldspars in Caleta nodules have anorthite (An = [atomic Ca/(Ca + Na + K)]) contents from An_6_ to An_87_ and are classified as bytownite, labradorite, andesine and anorthoclase (Fig. S8).

In Fasnia samples, clinopyroxenes range from 8.0 to 15.4 wt% MgO (Fig. S7), with Mg# between 53.5 and 86.0. Feldspars range from An_10_ to An_88_ and are similarly classified as bytownite, labradorite, and andesine. The Fasnia nodules contain clinopyroxenes ranging from high-Al titanaugite (Mg# 72–76) in ultramafic nodules to low-Al, Fe-rich green cores (Mg# 56–65) and Al-poor clinopyroxenes (Mg# ~76) in felsic nodules. Plagioclase compositions span An_44_ to An_88_, with sieve textures observed in some gabbroic nodules. Amphiboles, including kaersutite, are present in hornblende-bearing gabbros.

San Juan nodules feature clinopyroxenes with green cores (visible in PPL), comparable to the type-2 Fe-rich titanaugite cores in Fasnia nodules. These clinopyroxenes have low TiO_2_ (< 2.5% TiO_2_; Fig. S7a) and MgO ranging from 11.5 to 14.5 wt%, with Mg# between 67.8 and 78.1 (Fig. S7). Feldspars have not been characterised in San Juan samples, and their analysis remains an avenue for future work; here we focused on mafic phases that provide the strongest constraints on storage and differentiation processes. In Morteros nodules, clinopyroxenes have Mg# ranging from 72.6 to 74.7, while feldspars range in composition from An_70_ to An_85_. Finally, in Gaviotas nodules, preliminary analyses show clinopyroxene MgO ranges between 12.1 and 13.0 wt%, with Mg# from 68.2 to 74.7. For Gaviotas feldspars, An content from An_70_ to An_85_. Morteros and Gaviotas feldspars are classified Marshall (1996) as bytownite, labradorite. Further work is required to fully characterise all the mineral phases across the eruptions, but this dataset provides a valuable foundation for understanding compositional trends and overall mineralogical variations (Fig. 9).

### Interstitial melts

**Fig. 10:**
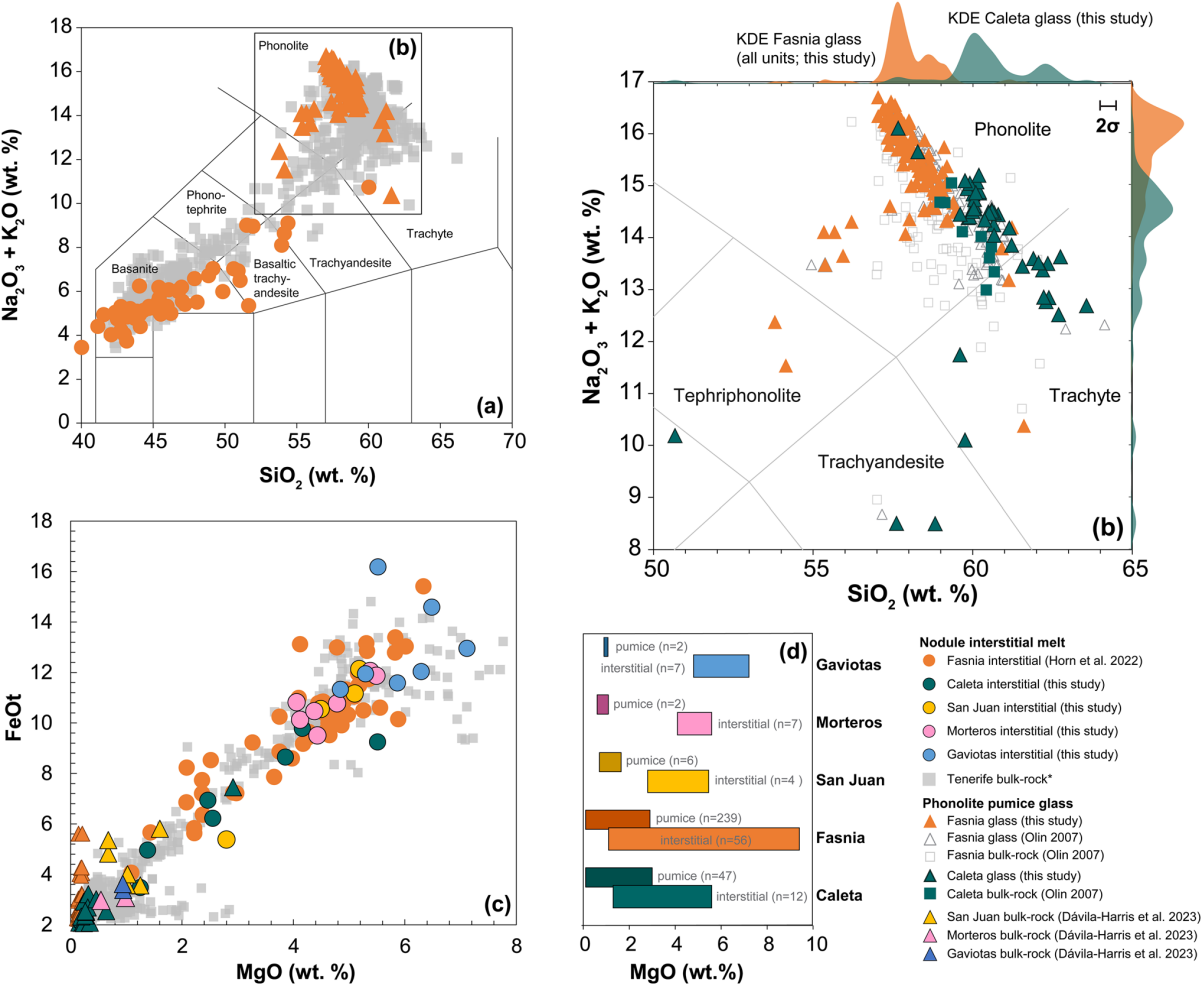
Interstitial melt, whole-rock and glass geochemical summary. **a** A total alkali-silica (TAS) plot (Le Bas et al. 1986) for Fasnia interstitial melts (Horn et al. 2022; circles) and Fasnia glass (this study; triangles). **b** TAS plot of Caleta and Fasnia glass and bulk-rock pumice data from this study and Olin (2007). Kernel density estimation (KDE) curves show the probability distribution of glass compositions from each eruption (only data from this study). **c** MgO versus FeO: Major elements interstitial melt compositions for all eruptions alongside co-existing pumice. (d) Bar graphs summarises the MgO (wt%) range for interstitial melts and pumice for each eruption unit (Gaviotas, Morteros, San Juan, Fasnia, Caleta), with data from this study, Olin (2007), Horn et al. (2022), Dávila-Harris et al. (2023). All the analyses presented in the graphs have been normalized to 100%. Error bars on graphs are plotted where analytical uncertainties are larger than the size of a data symbol and represent 2 standard deviations of replicate analyses of MPI-DING StHs6/80-G glasses, 2σ for SiO_2_ is 0.5%, Na_2_O (0.3%) and K_2_O (0.06%). Literature whole-rock and glass data (grey symbols; *n* = 716) are compiled from GeoROC and EarthChem databases; Wolff (1985), Palacz and Wolff (1989), Ablay et al. (1995, 1998), Bryan et al. (1998; 2002; 2006), Neumann et al. (1999, 2000), Wolff et al. (2000), Edgar et al. (2002), Gottsmann et al. (2001), Rodriguez-Badiola et al. (2006), Olin (2007), Clay et al. (2011), Wiesmaier et al. (2011), Deegan et al. (2012), Sliwinski et al. (2015), Di Roberto et al. (2016), Dávila-Harris et al. (2023).

**Fig. 11:**
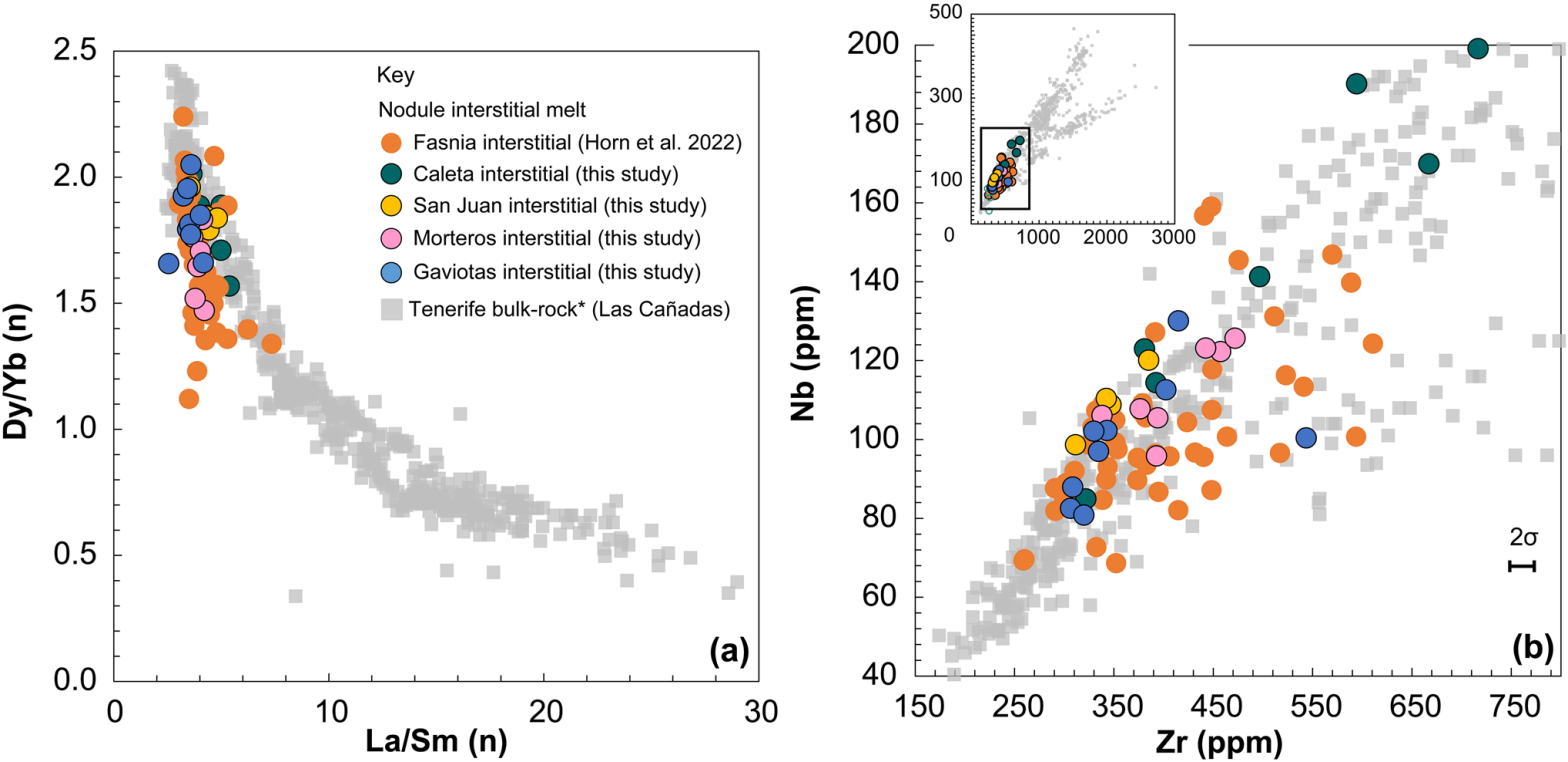
Interstitial melt trace element summary **a** REE of interstitial melt and groundmass separates [La/Sm]_n_ and [Dy/Yb]_n_, as examples of the relationship between LREE/MREE and MREE/HREE. Chondrite normalization factors (n) are from Evensen et al. (1978). **b**: Nb-Zr plots all Tenerife data, with all eruptions interstitial melt and for Gaviotas (1.84 Ma), Morteros (∼1.75 Ma), San Juan (1.50 Ma), Fasnia (0.312 Ma) and Caleta (0.22 Ma) juvenile nodules (circles). Literature whole-rock (grey symbols; *n* = 716) are compiled from GeoROC and EarthChem databases; Bryan et al. (2006), Neumann et al. (1999, 2000), Thirlwall et al. (2000), Wolff et al. (2000), Gottsmann et al. (2001), Rodriguez-Badiola et al. (2006), Olin (2007), Deegan et al. (2012), Sliwinski et al. (2015), Turner et al. (2017), Di Roberto et al. (2016), Dávila-Harris et al. (2023).

Major and trace element data for interstitial melt from Caleta, San Juan, Morteros and Gaviotas eruptions are presented in the Supplementary Information (S1), Figs. 10 and 11. The MgO content of the interstitial melt shows considerable variability between eruptions (Table 3; Fig. 10c). For Caleta, MgO ranges from 1.3 to 5.5 wt% (*n* = 12). The Fasnia melt exhibits a broader MgO range of 1.1 to 9.2 wt% (*n* = 56), while San Juan has a more restricted MgO range of 2.8 to 5.2 wt% (*n* = 4), but this has a much smaller sample size in comparison. In the Morteros, MgO ranges from 4.1 to 5.5 wt% (*n* = 7), and the Gaviotas ranges between 4.8 and 7.1 wt% (*n* = 7). Major element composition of interstitial melt from Caleta, San Juan, Morteros and Gaviotas nodules fall within the range of those from Fasnia (Fig. 10a), with Fasnia interstitial melts extending from basanite to trachyte. The MgO content of Tenerife interstitial melts is wide, with all of the interstitial melt samples having an average MgO concentration of 4.4 wt% (*n* = 81). Co-existing pumices from the same eruption units consistently have lower MgO contents than nodules in the same unit, with an average pumice MgO of 0.4 wt% across all of our samples (*n* = 368; Table 3; Fig. 10d).

**Fig. 12:**
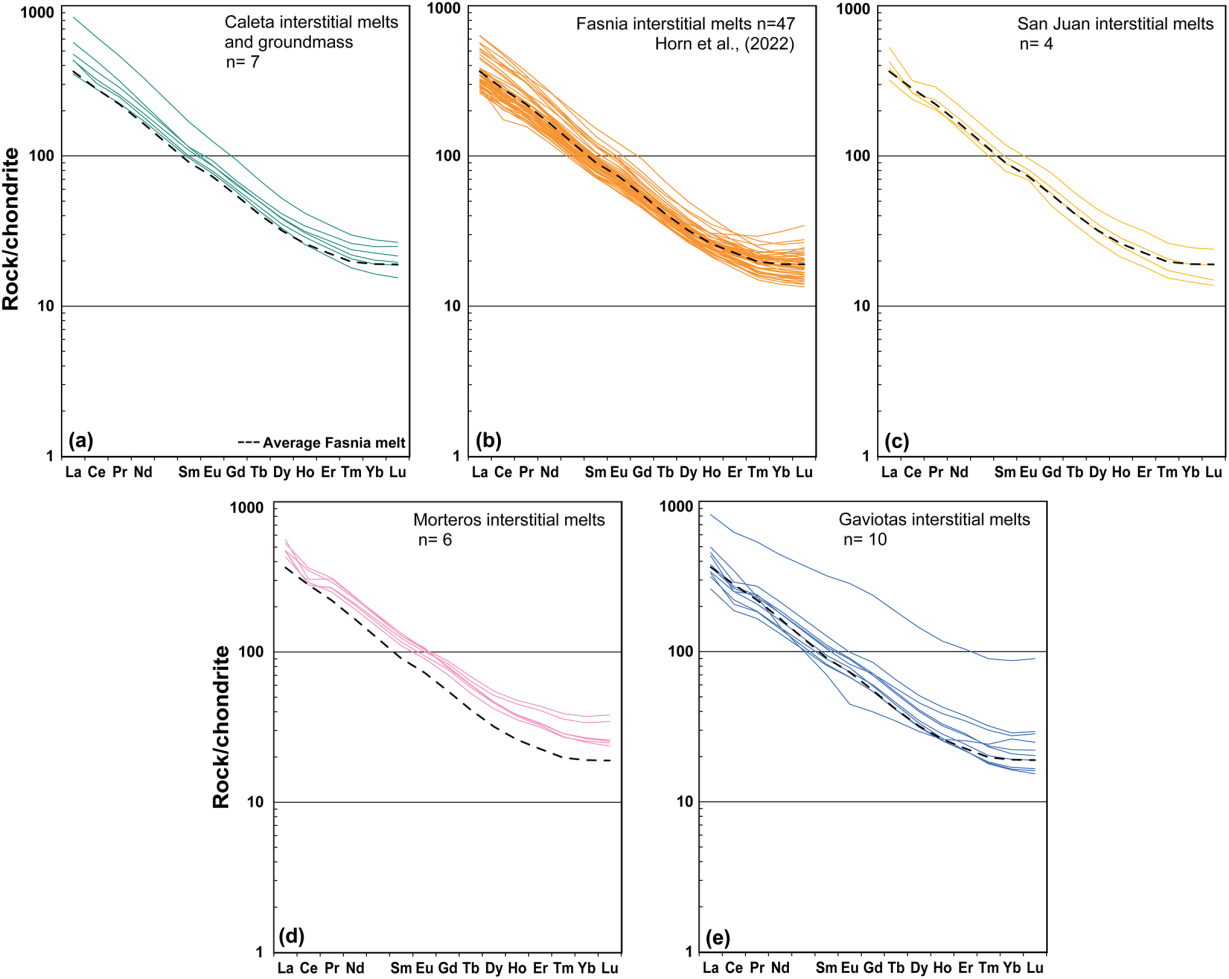
Chondrite-normalized REE patterns for Tenerife juvenile nodule interstitial melt separates, split by eruption. The average REE profile Fasnia interstitial melts is shown in each plot as a black dashed line (Horn et al. 2022). Chondrite normalization factors are from Evensen et al. (1978).

The Zr and Nb concentrations vary systematically between eruptions (Fig. 11b; Supplementary Data S1). In the interstitial melts Zr values are lowest in the Fasnia eruption (260–610 ppm; Horn et al. 2022) and reach the highest levels in Caleta (up to ∼950 ppm), while Nb follows the same trend, ranging from 70–160 ppm in Fasnia to nearly 200 ppm in Caleta. The San Juan, Morteros, and Gaviotas melts show intermediate values (310–540 ppm Zr; 80–125 ppm Nb). Three interstitial melt samples from Caleta have higher Nb/Zr than other interstitial melt samples (Fig. 11b), with two of the samples able to be linked to visibly mixed melts observed in thin section (Fig. S1). All other trace elements for interstitial melts from Caleta, San Juan, Morteros and Gaviotas nodules also fall within the same range as those from Fasnia (Fig. 11). Interstitial melts from Caleta, Fasnia, San Juan, Morteros, and Gaviotas have enriched HREE to LREE patterns, typical of Tenerife basaltic magmas (Wolff 2000). Chondrite-normalized REE elements for each nodule’s interstitial melt are also comparable between eruptions (Fig. 11) and [La/Sm]_n_ (LREE/MREE) and [Dy/Yb]_n_ (MREE/HREE) from these eruptions are, again, comparable with those from Fasnia eruption (Fig. 12), albeit with less variation. Overall, the interstitial melts show little fractionation compared to the phonolite pumice from the same eruptions, as already indicated in previous works (Horn et al. 2022; Dávila-Harris et al. 2023).

### Pumice glass

Table 4 summarises the average major element glass compositions for the Caleta and Fasnia units from this study (the complete dataset is provided in Supplementary Information S2). For comparison, whole-rock pumice data from the San Juan, Morteros, and Gaviotas eruptions are taken from Dávila-Harris (2023). It was not possible to obtain glass data for the older eruptions in this study due to age-related alteration, which has compromised the preservation of fresh glass.

Pumice glass compositions across the eruptions are consistently trachytic to phonolitic, but show subtle differences between eruptions (Fig. 10b; Table 4). Caleta glasses average ~ 60 wt% SiO₂ with lower total alkalis (~ 13.8 wt%), whereas Fasnia glasses are slightly less silicic (~ 58 wt% SiO₂) but more alkaline (~ 15.5 wt%). Within Fasnia, upper and lower sub-units differ subtly in SiO₂ and alkali contents, consistent with previous observations (Edgar et al. 2017). Whole-rock pumice data from San Juan, Morteros, and Gaviotas (Dávila-Harris et al. 2023) show broadly comparable trachytic compositions, but with slightly higher MgO contents (0.8–1.0 wt%) than Caleta or Fasnia. The pumice glass MgO contents are consistently lower than those of the interstitial melts (Fig. 10d; Table 3). Glasses from Caleta and Fasnia show overlapping MgO ranges (0.1–2.9 wt%), with moderate heterogeneity, consistent with earlier observations of intra-eruptive variability in Fasnia pumice (Olin 2007; Table 4). When compared directly with interstitial melts, pumice glasses extend to lower MgO values, although there is some overlap between the two datasets (Fig. 10d). Whole-rock pumice from older eruptions shows broadly similar MgO contents: San Juan (0.7–1.6 wt%), Morteros (0.6–1.0 wt%), and Gaviotas (0.9 wt%) (Dávila-Harris et al. 2023).

## Discussion

### Magma reservoir diversity

#### Temporal snapshots of the Tenerife mush system

The microcrystalline or glassy interstitial material observed in juvenile nodules indicate that the mush was supra-solidus at the time of eruption (modal average ~ 25% across eruptions, ranging from 19.4 to 26.4 vol% for melt-bearing nodules; Table 3). Juvenile nodules therefore represent fragments of active crystal-rich entrained immediately prior to caldera-forming eruptions (Stock et al. 2012; Horn et al. 2022). These nodules provide valuable “snapshots” of the mush reservoir during eruption, consistent with the basanite crystalline mush reservoir hypothesised beneath Tenerife (Sliwinski et al. 2015). Seismic tomography reveals low-velocity, high Vp/Vs anomalies at ~ 5 km depth beneath Las Cañadas, consistent with the presence of a phonolitic reservoir (Koulakov et al.^ 2023^), while deeper attenuation anomalies indicate hot, partially molten crustal rocks near the Moho (Prudencio et al. 2015). Gravity and magnetotelluric data further identify dense intrusive cores surrounded by low-density, low-resistivity bodies interpreted as melt and fluid-rich zones (Ablay and Kearey 2000; Araña et al. 2000; Pous et al. 2002; Gottsmann et al. 2008; Piña-Varas et al. 2018). Together, these observations support a vertically extensive, mush-dominated reservoir system beneath Tenerife. Across five eruptions spanning ~ 1.8 to 0.22 Ma (Gaviotas to Caleta; Table 1), the persistence of similar nodule textures and interstitial melts suggests that a mush reservoir has been a stable and integral component of the Tenerife system for ~ 1.8 million years.

Despite this long temporal range, the nodules show limited between-eruption variability. Interstitial melt major and trace element ratios are broadly comparable across all five eruptions (Figs. 10 and 11), even though individual eruptions display internal compositional heterogeneity. Mineral assemblages are repeatedly dominated by clinopyroxene, plagioclase and amphibole with only local variations (e.g., layering; Fig. 4e) and the same lithologies recurring in all studied eruptions (Fig. 5; Table 2). Superimposed on this broad stability are localised heterogeneities (e.g., phases in dis-equilibrium shown through sieve textures, Fig. 6a; intra-crystal chemical zoning; Stock et al. 2012; Horn et al. 2022) and a range of interstitial melt compositions (e.g., interstitial melt MgO ranges in Caleta: 1.3–5.5 wt% and Fasnia: 1.1–9.2 wt%; Table 3; Fig. 10d). These features indicate dynamic melt-crystal interactions and pockets of melt evolving along slightly different paths before fragmentation and eruption, offering snapshots of short-term and crystal-scale compositional diversity within the mush (Suh et al. 2003; Solano et al. 2012; Jackson et al. 2018; Ubide et al. 2024). However, the overall mineralogical consistency and the similarity of interstitial melts across nodules from different eruptions suggest that the overall temperature, pressure, and composition were maintained over extended timescales.

Thermobarometric estimates for Tenerife’s phonolitic, tephritic and basanitic reservoirs, indicate storage at ~ 825–890 °C and ~ 130 MPa for phonolite, ~ 1050 °C and 410–450 MPa for deeper tephritic magmas (Andújar et al. 2008; González-García et al. 2022), and ~ 1050–1150 °C at 300–600 MPa for basanite storage in the lower to mid-crust (Ablay 1997; Neumann et al. 1999). These conditions are consistent with a vertically extensive mush system, where melt is intermittently replenished and redistributed while crystal-dominated regions remain largely unerupted over long timescales (Bachmann et al. 2007; Cashman et al. 2017).

The longevity of this mush system is likely best explained by its vertical extent and repeated replenishment from a long-lived source. Low-flux OIB settings generally favour deeper melt retention rather than sustained mid-crustal melt storage (Longpré et al. 2008; Ubide et al. 2022; Gleeson et al. 2023), meaning persistent mushes can be repeatedly reheated or flushed by new inputs of mafic melts. The juvenile nodules provide physical evidence of this mafic melt percolating through the mush immediately prior to eruption. Over million-year timescales, slow but recurrent recharge can maintain temperatures near the solidus and preserve consistent mineral assemblages (Annen and Sparks 2002; Ducea et al. 2015; Lipman and Bachmann 2015; Menand et al. 2015; Kaiser et al. 2017; Cruden and Weinberg 2018). Reactive melt flow further contributes to this stability by maintaining a dynamic equilibrium between melt input and extraction, buffering the mush composition despite episodic recharge, preserving a complex yet compositionally stable mush (Marsh 1996; Parmigiani et al. 2014; Annen et al. 2015; Taylor et al. 2020; Gleeson et al. 2023).

### Changes in magma chemistry with time

The geochemical evolution of Tenerife’s magmatic system over the past ~ 1.8 Myr highlights the interplay between long-term stability in mafic mush reservoirs and episodic variability in phonolitic melts. Dávila-Harris et al. (2023) documented systematic changes in incompatible elements such as K, Zr, Nb, and Rb, which become progressively enriched up stratigraphy. For example, Zr/Nb ratios evolve over time, dividing Tenerife’s eruptive history into distinct geochemical groupings (Wolff et al. 2000; Edgar et al. 2007). These groupings reflect cumulative fractional crystallisation, episodic mantle-derived inputs, and crustal assimilation during magma evolution (Olin 2007; Wolff et al. 2015; Dávila-Harris et al. 2023). These trends are particularly evident in eruptions such as Gaviotas (1.84 Ma) and Mocan (1.49 Ma), where progressive increases in Fe₂O₃ and decreases in Al₂O₃ suggest differences in the proportions of crystallising phases such as clinopyroxene and plagioclase. Dávila-Harris et al. (2023) further identifies timescales for these chemical changes, showing that they occur over 100,000-200,000 years, linked to shifts in magma storage depths or lateral position in the crust and recharge events. This timescale aligns with the episodic nature of mantle input and magmatic differentiation processes observed in other studies (Hawkesworth et al. 2004; Peate and Hawkesworth 2005; Stracke 2012; Cooper 2019; Edmonds et al. 2019).

In contrast to evolving phonolite magmas, the interstitial melts in nodules retain consistent compositions through the five eruptions (~ 1.8 Myr). Average MgO contents are ~ 4.4 wt% (range: 1.3–9.2 wt%, Table 3), and trace element ratios (e.g., Dy/Yb; Zr/Nb, Fig. 11), and Nb-Zr concentrations are comparable to the Las Cañadas mafic lavas (Wolff et al. 2000). This points to a chemically buffered mantle source feeding a stable mafic mush on million-year timescales (Sparks et al. 2019; Winslow et al. 2022). By contrast, pumice glasses from Caleta (221 ka) and Fasnia (312 ka) differ (Caleta higher SiO₂, lower total alkalis than Fasnia; see Table 4). These differences on shorter timescales imply distinct felsic-reservoir evolutionary paths controlled by variable fractional crystallisation and assimilation over timescales of ~ 90 kyrs (Ablay et al. 1998; Wolff et al. 2015; Dávila-Harris et al. 2023). This variability likely reflects the compartmentalised and possibly laterally variable mush architecture beneath Tenerife, where felsic reservoirs evolve independently from the long-lived mafic mush. The absence of intermediate compositions suggests that structural and rheological barriers limit large-scale mixing between basanite mush zones and overlying felsic reservoirs, with interaction occurring only locally, as recorded by mingled pumices (e.g., Wolff 1985; Olin 2007; Figs. 6d, S1c). In this crystal-rich framework, the mafic mush provides a stable, chemically buffered base, while discrete felsic pockets undergo episodic differentiation, recharge, and mingling, producing the observed short-term variability.

**Fig. 13:**
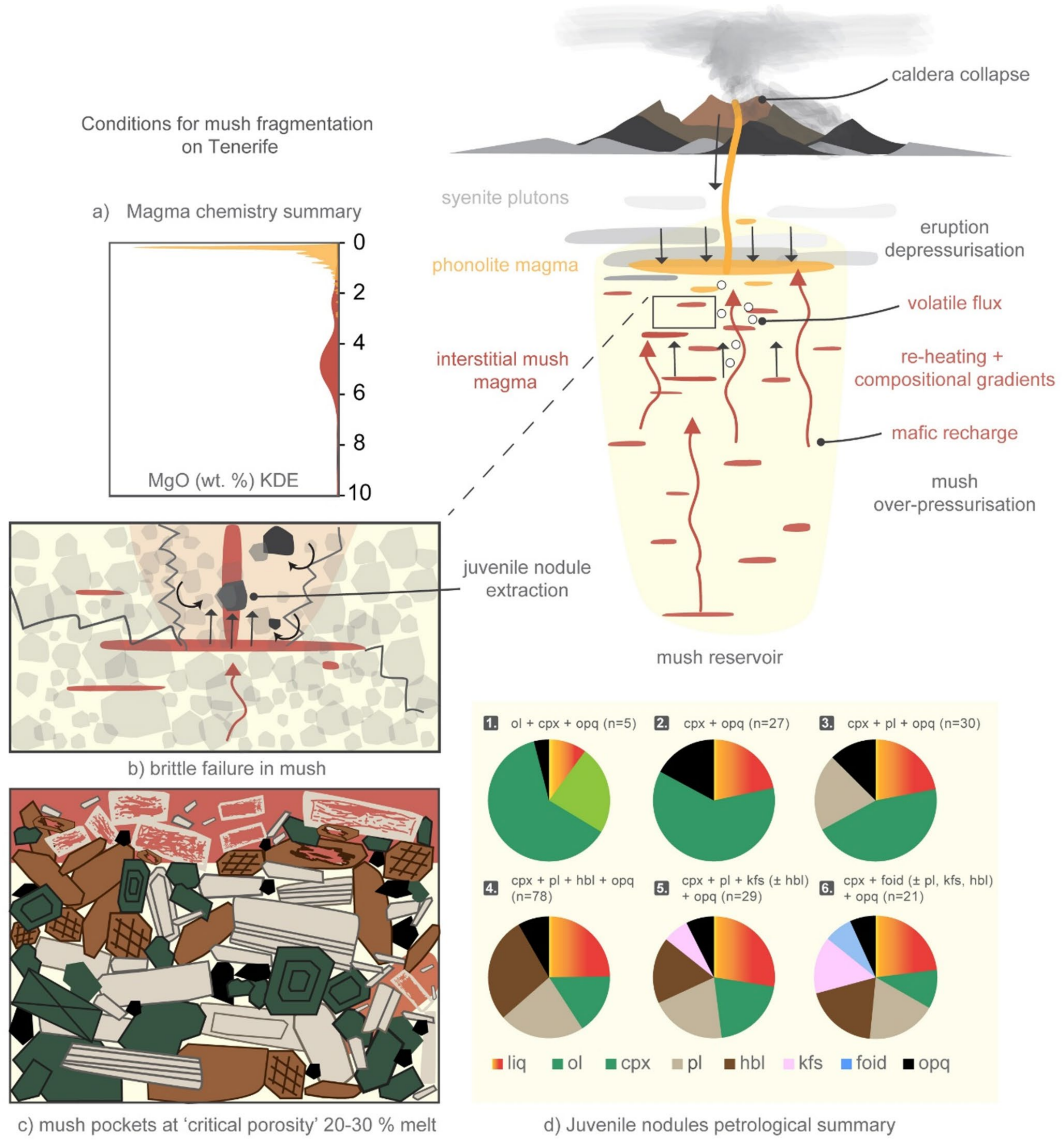
Schematic representation of the Tenerife magmatic system and conditions for mush fragmentation. Simplified model of the Las Cañadas edifice illustrating the long-lived basanite mush reservoir and its relationship with the phonolite reservoir. **a** Magmatic liquid chemistry summary showing the probability distribution (Kernel Density Estimates; KDE) of MgO (wt%) for all eruption liquids combined, highlighting the compositional bimodality between phonolitic and interstitial basanite magmas. **b** depiction of brittle failure and overpressurisation within the mush reservoir during eruption, where volatile flux and reheating generates transient pressure gradients enabling disaggregation and nodule entrainment. **c** cartoon crystal framework. **d** a petrological summary of juvenile nodules, detailing the proportion and distribution of major mineral phases for all the eruptions combined (liq = interstitial melt; ol = olivine; cpx = clinopyroxene; pl = plagioclase; hbl = hornblende; kfs = K-feldspar; foid = feldspathoid; opq = oxides) across six lithological groups: (1) wehrlite, (2) clinopyroxenite, (3) gabbro, (4) pyroxene-hornblende gabbro, (5) monzodiorite/monzonite, and (6) feldspathoid monzosyenite. Arrows in the central schematic indicate the transfer of interstitial melt, mafic recharge, volatile migration, and extraction of mush fragments during explosive eruptions.

The absence of intermediate compositions (e.g., tephriphonolites or trachyandesites) in both interstitial melts and pumice glasses from these Cañadas eruptions underscores the bimodal nature of this part of Tenerife’s magmatic system. By contrast, intermediate compositions are observed in the younger Teide–Pico Viejo complex, particularly at Pico Viejo (Ablay et al. 1998; Andújar et al. 2013). This difference may reflect a focus in this study, and much of the existing work, on explosive, phonolite-dominated eruptions of the Cañadas edifice, whereas effusive and mixed-style eruptions at Teide-Pico Viejo have produced a broader compositional spectrum. Alternatively, the contrast could reflect an evolutionary change in the system, with the younger Teide-Pico Viejo complex tapping less isolated reservoirs or allowing more efficient interaction between mafic and felsic magmas. Structural and rheological barriers within the crust could inhibit significant mixing between Las Cañadas mafic and felsic reservoirs, as suggested by geophysical studies (Martí et al. 1997; Carracedo et al. 2007; Carracedo and Perez-Torrado 2013). This compartmentalisation aligns with observations from other ocean island systems, such as Ascension and the Galápagos, where primitive melts are retained at depth, while more evolved magmas are erupted (Geist et al. 1998; Chamberlain et al. 2019). For instance, Ubide et al. (2022) highlight that MgO contents of erupted melts often cluster around 5 wt% due to fractional crystallisation and retention of primitive compositions in deep reservoirs, consistent with the data presented here (Fig. 13; Table 3).

REE trends provide further evidence of the stability of the mafic mush reservoir. Interstitial melts exhibit consistent REE ratios ([La/Sm]_n_ and [Dy/Yb]_n_) across eruptions (Figs. 11 and 12), again comparable to basanitic lavas from Tenerife (Wolff et al. 2015; Horn et al. 2022). These patterns reflect a uniform mantle-derived signature, consistent with observations of geochemical consistency in basanitic magmas across Tenerife and other ocean island systems (Wolff et al. 2015; Ubide et al. 2022). In contrast, phonolitic magmas on Tenerife exhibit variability in REE patterns that primarily reflects differentiation processes such as fractional crystallisation and feldspar assimilation within felsic reservoirs, rather than significant recharge of less-evolved melts. Positive Eu anomalies and variations in [La/Sm]_n_ and [Dy/Yb]_n_ ratios observed in phonolitic magmas are consistent with feldspar fractionation and the melting of syenitic cumulates or feldspar-rich crystal mushes (Wiesmaier et al. 2012; Wolff et al. 2020; Dávila-Harris et al. 2023; Dorado et al. 2023). Isotopic data provides additional insights into these processes. Pb isotopic evolution observed by Taylor et al. (2020) reveals temporal changes in both mafic and felsic magmas over ~ 1.8 Ma, driven by shifts in mantle plume composition. While the mafic system remains isotopically and chemically stable, the phonolitic reservoirs exhibit greater variability, reflecting processes such as fractional crystallization, assimilation and episodic recharge (Ablay et al. 1998; Sliwinski et al. 2015; Turner et al. 2017; Cas et al. 2022). These findings support the long-term stability of the mafic reservoir contrasted with the episodic variability of felsic magmas. Although Pb isotope data demonstrate that the Canary mantle plume undergoes chemical “pulses” on 0.3-1 Myr to multi-Myr timescales (Taylor et al. 2020), the mafic reservoir beneath Tenerife records remarkably consistent major and trace element signatures. This suggests that large, long-lived mush zones buffer short-term mantle variability, producing relatively ‘stable’ erupted compositions, while smaller felsic reservoirs evolve more variably in response to fractional crystallisation, assimilation, and recharge.

The full range of crystallisation stages in the alkaline system with varying mineral proportions (Fig. 5) reveals the potential for diverse magma compositions and conditions coexisting within the system. This highlights the complex interplay of magmatic processes shaping Tenerife’s chemical evolution. A common feature across our studied eruptions is the coexistence of basanite and phonolite magmas in the Tenerife magmatic reservoir prior to large explosive eruptions: interstitial melts are almost exclusively basanitic, whereas the pumice in erupted ignimbrite and fall deposits are highly fractionated trachyte-phonolite (Fig. 10), with minimal evidence of intermediate tephriphonolitic or trachyandesitic compositions in the nodules. The compositional consistency of interstitial melts across five sampled eruptions suggests that the mafic mush reservoir has been chemically stable during these discrete events. Conversely, temporal variability in felsic reservoirs highlights the dynamic nature of Tenerife’s magmatic evolution. This duality likely reflects the compartmentalised architecture of Tenerife’s magmatic system, where reservoirs evolve independently over centennial to Myr-scale timescales (Sliwinski et al. 2015; Taylor et al. 2020; Cas et al. 2022; Dávila-Harris et al. 2023). Notably, changes in the primary melt source, as suggested by Pb isotopic variability in mantle-derived magmas (Glazner et al. 2004; Deegan et al. 2012; Taylor et al. 2020), may influence the broader magmatic system. By integrating geochemical trends with the timescales proposed by Dávila-Harris et al. (2023), this study emphasises the bimodality of Tenerife’s magmatic system: a stable, long-lived mafic mush reservoir and episodically variable felsic reservoirs shaped by cycles of mafic recharge, crystallisation, assimilation, and changes in storage geometry. This contrast highlights how Tenerife’s magmatic architecture simultaneously buffers mantle variability in the mafic system while recording short-lived, eruption-relevant processes in the felsic reservoirs.

### Evidence for melt channelisation and ascent

Relative to Fasnia, Caleta interstitial melts include a larger fraction of intermediate compositions (lower MgO, higher Zr; Fig. 11) and several thin sections show mixing textures (Fig. S1). A subset of nine Caleta nodules bear a distinct exterior coating with different mineralogy/texture to the nodule cores (Fig. 8). The coating lacks grain-grain contacts or alignment within the groundmass, and its higher average melt fraction (~ 45%) suggests a fragmented, remobilised mush layer close to the rheological mush-magma transition (cf. Sparks et al. 2019). These layers were not observed in any of the nodules analysed in the Fasnia (Horn et al. 2022) or the other three eruptions in this study. Based on the absence of grain-grain contacts or grain alignment within the groundmass in this coating, we suggest it represents a fragmented, remobilised mush with a higher average melt proportion of ~ 45%. The crystal fraction within these melt-rich layers is close to the rheological boundary between mush and magma (Sparks et al. 2019). We interpret these coatings as transient, melt-rich channels (dyke/sill-like conduits) that transported melt, crystals, xenoliths and more crystal-rich nodules dislodged from the mush but not fully disaggregated (Klügel 1998). In this scenario, channelised melts act as a carrier liquid and upon evacuation and quenching, crystal-rich coatings accreted onto nodule exteriors. Short-lived pathways are expected in dynamic mushes, and similar processes have been observed in volcanic systems where transient pathways develop under dynamic conditions (Mollo and Hammer 2017; Sparks and Cashman 2017; Biggs and Annen 2019; Edmonds et al. 2019). Melt flow within mush zones creates temporary pathways for crystal-melt transport under pressure gradients, thermal instabilities, or reheating events (Spiegelman and Kelemen 2003; Lissenberg et al. 2013; Solano et al. 2014). Petrological evidence of these pathways on a macroscopic scale is evidenced in the Fasnia nodules (Fig. 5d of Horn et al. 2022). The formation of these pathways likely reflects transient pressure gradients or thermal instabilities within the mush reservoir, driven by gas expansion or rapid depressurisation (Druitt and Sparks 1984; Métrich and Wallace 2008; Edmonds et al. 2019; Woods and Stock 2019). These mechanisms are consistent with the role of dykes and sills as ephemeral melt pathways that facilitate material transport within vertically extensive magmatic systems (Cashman et al. 2017). The rarity of coatings across the dataset may indicate that melt channelisation was highly episodic and/or rarely preserved. In the Caleta eruption, however, the preservation of an additional melt-rich layer on nodule margins, together with subtle differences in pumice glass compositions (Fig. 10b), points to distinct pre-eruptive conditions. These may reflect stronger or more sustained mafic recharge, enhanced remobilisation of mush domains, or greater melt-mush interaction relative to other juvenile nodule-bearing eruptions.

#### Fragmenting reservoir on eruption

Lithic breccia horizons in Tenerife’s phonolitic ignimbrites provide crucial evidence for high energy eruptive phases, reflecting a combination of conduit fragmentation, volcano-tectonic processes such as roof collapse and vent widening (Walker 1985; Bryan et al. 1998; Soriano et al. 2006), and in some cases fragmentation of deeper mush reservoirs during explosive eruptions. For example, the Ravelo unit of the Fasnia eruption shows evidence of pulses of fragmentation, which likely reflect episodic increases in pressure or conduit destabilisation during the eruption (Druitt and Sparks 1984; Edgar et al. 2017; Caricchi et al. 2021). In addition, juvenile nodules are concentrated in the coarsest breccia facies, particularly in Caleta and Fasnia deposits, suggesting that mush-bearing reservoir fragments were entrained during the most intense phases of these eruptions (Pittari et al. 2006). Notably, in Horn et al. (2022) we also reported nodules in fall deposits, implying that mush fragmentation might begin from the eruption onset, when the system is primed for high degrees of instability. Interestingly, juvenile nodules are not ubiquitous across Tenerife’s ignimbrite deposits. For example, the Poris and Abrigo eruptions (i.e., Pittari et al. (2008); Stock et al. (2012) contain fewer nodules, and many of the lithics lack juvenile microcrystalline groundmass indicative of a pre-eruptive mush. In particular, the Abrigo eruption, which is among the largest recorded eruption on Tenerife (Martí et al. 1994; Ablay and Kearey 2000), contains abundant lithics (Pittari et al. 2008), yet juvenile nodules are scarce. This discrepancy suggests that even large eruptions do not necessarily mobilise mush, highlighting an important area for future work. Understanding why large-volume eruptions lack juvenile nodules would help refine our models of mush mobilisation during caldera-forming events (cf. Pittari et al. 2008; Holness et al. 2019). This variability and overall scarcity in nodule occurrence imply that mush fragmentation and entrainment require specific structural and rheological conditions within the reservoir. The mechanical conditions under which Tenerife’s crystal mush fragmented remain poorly constrained but likely reflect a combination of thermal, mechanical, and structural processes. Fragmentation of a crystal-rich mush may occur when volatile exsolution or magma recharge generates local overpressure, or when the system crosses a rheological threshold between ductile and brittle behaviour at melt fractions of ~ 30–40 vol% (Humphreys et al. 2025). In Tenerife, such transitions may have been triggered by caldera fault movement or pressure gradients during phonolite withdrawal, causing brittle failure of the mush and entrainment of juvenile nodules. These processes emphasise the dynamic feedback between reservoir pressurisation, structural control, and eruption initiation, and represent key targets for future experimental and modelling work.

At a crystal fraction of 50–60 vol%, mush is considered rheologically locked and uneruptable (Marsh 1981), yet on average the nodules are even more crystalline (average ~ 75 vol% solids). This creates a paradox: the nodules should be uneruptable, yet they are found in several ignimbrites. Their occurrence in lithic-rich breccias of Caleta, Fasnia, San Juan, Morteros, and Gaviotas indicates these eruptions provided the exceptional conditions required to destabilise and disaggregate an otherwise rigid mush framework (Humphreys et al. 2025). In particular, these eruptions appear to have tapped melt-rich reservoirs nearing “critical porosity” thresholds of between 20 and 30% melt (Marsh 1981, 2015; Bachmann and Bergantz 2008b), where the mush transitions from rigid to mobile, making it more susceptible to disaggregation under stress. The melt fractions we measure vary within single nodules (heterogeneous groundmass distribution), but eruption-averaged modal melt abundances cluster near ~ 25% (19.4–26.4%; Table 3), consistent with this critical porosity window. At such states, the mush is prone to disaggregation when pressure gradients, driven by gas expansion or influx, exceed yield strength and aid fragmentation, as inferred in other explosive systems (Spieler et al. 2004; Toramaru and Miwa 2008; Cashman and Sparks 2013; Cassidy et al. 2018; Giordano et al. 2024). This is consistent with experimental and numerical studies showing mush destabilisation occurs under volatile flux, deformation, and reheating (Huber et al. 2011; Parmigiani et al. 2014; Pistone et al. 2015, 2017; Humphreys et al. 2025). Compositional or chemical gradients, particularly at basanite-trachy-phonolitic interfaces, created during magma recharge events (Bindeman 2005), can further promote brittle failure in mush zones (Fernández and Castro 2018; Holness et al. 2019; Haag et al. 2024). Compositional profiles across Caleta plagioclase show narrow rim zones (< 40 μm) that, using diffusion chronometry, are estimated to have grown over ~ 7–132 days prior to eruption (Larsen 2005; see Stock et al. (2012), supporting late-stage reheating and mixing immediately before evacuation of the mush.

In addition to these internal factors, comparisons with other large-volume ignimbrites have shown that crystal fragmentation can occur in response to over pressurisation of the mush (Huber et al. 2011; van Zalinge et al. 2018). Evidence from other caldera-forming systems, such as those in the Central Andes, underscores the role of external forces such as rapid decompression on caldera collapse or conduit destabilisation, which can exacerbate magma fragmentation and mobilise crystal-rich magmas (Druitt et al. 2002; van Zalinge et al. 2018; Okumura et al. 2019). Together, these studies suggest that the extraction of crystal-rich mushes during Tenerife’s explosive eruptions likely involves a combination of internal and external processes, where pressure changes, melt content, and external decompression during caldera collapse collectively contributed to fragmentation and mush mobilisation (Fig. 13). Although thermobarometric estimates for tephritic magmas place storage near the base of the crust (~ 12–15 km), it is not necessary to invoke direct entrainment of nodules from such depths. Instead, nodules likely represent fragments of mush zones located at mid-crustal levels, adjacent to phonolitic-tephritic reservoirs. Their interstitial melts are basanitic, periodically flushed by more-primitive melts rising from depth. In this scenario, the nodules preserve the crystal framework of mush systems, while their melt components record ongoing recharge from deeper sources (Neumann et al. 1999; Wolff et al. 2000; González-García et al. 2022). The nodules document parts of the mush reservoir that were normally uneruptable but were temporarily destabilised and mobilised by a combination of pressure gradients, melt fluxes, and eruption-triggered disaggregation. Entrainment into phonolitic ignimbrites reflects the complex and restricted occurrence of juvenile nodules, highlighting their importance as rare but invaluable records of Tenerife’s mush reservoirs.

## Conclusions

The primary finding of this work, alongside Horn et al. (2022), is the provision of new physical evidence for the presence of an enduring crystal mush reservoir beneath Tenerife, that has existed for at least the past ~ 1.8 Myr. Juvenile nodules erupted during multiple Plinian and caldera-forming events preserve average melt fractions of ~ 25%, placing them near the rheological mush-magma transition, consistent with the melt segregation and classification of mush systems described by Hildreth (1981) and Bachmann and Bergantz (2004).

The basanite mush reservoir appears to have remained chemically stable over million-year timescales, with interstitial melts showing consistent major and trace element compositions across eruptions, indicating repetitive processes within crustal reservoirs. A common feature across the five eruptions studied is the coexistence of both basanite and phonolite magmas in the Tenerife magmatic reservoir prior to eruption. In contrast, phonolitic magmas erupted in the same events are more variable, reflecting episodic fractional crystallisation, assimilation, and recharge. Despite some evidence of magma mingling, most interstitial melt compositions in the mush reservoir are chemically unrelated to the phonolite found in the corresponding ignimbrite deposits. This highlights a strongly bimodal system, where stable basanite mush underpins a more dynamic, independently evolving felsic reservoir.

Importantly, the occurrence of mush-bearing nodules is rare and restricted to certain eruptions. They are not present in all Tenerife lithic-rich ignimbrites, implying that mush disaggregation and entrainment only occur under specific eruptive and tectonic conditions. Indeed, to our knowledge they are not found in similar caldera-forming systems globally. Their preservation in ignimbrites provides unique “snapshots” of Tenerife’s mush system at the point of eruption, revealing both its long-term stability and its capacity for sudden mobilisation.

Juvenile nodules thus provide direct evidence for a crystal mush framework beneath Tenerife, in which igneous systems are dominated by largely uneruptible, crystal-rich material. Collectively, these findings refine our understanding of Tenerife’s reservoir structure and demonstrate how mafic mushes and episodic felsic reservoirs interact to drive bimodal behaviour that is perhaps characteristic of ocean island volcanoes.

## Supplementary Information

Below is the link to the electronic supplementary material.

Supplementary Material 1

Supplementary Material 2

Supplementary Material 3

## Data Availability

Supplementary tables and figure source files are included with this manuscript. All underlying analytical data from the nodule samples are openly available through the doctoral thesis of Horn (2023), (University of Southampton) https://eprints.soton.ac.uk/477058/ and the open-access publication Horn et al. (2022).
