# Supplementary material for: Melt-bearing nodules from Tenerife reveal magma reservoir diversity prior to caldera-forming eruptions
Source: Contrib Mineral Petrol. 2026 Apr 28;181(5):39. doi: 10.1007/s00410-026-02302-3 (PMC13121400; doi:10.1007/s00410-026-02302-3)
Supplement: Supplementary file 3 — Supplementary Material 3 [file 410_2026_2302_MOESM3_ESM.docx]

# Supplementary Figures


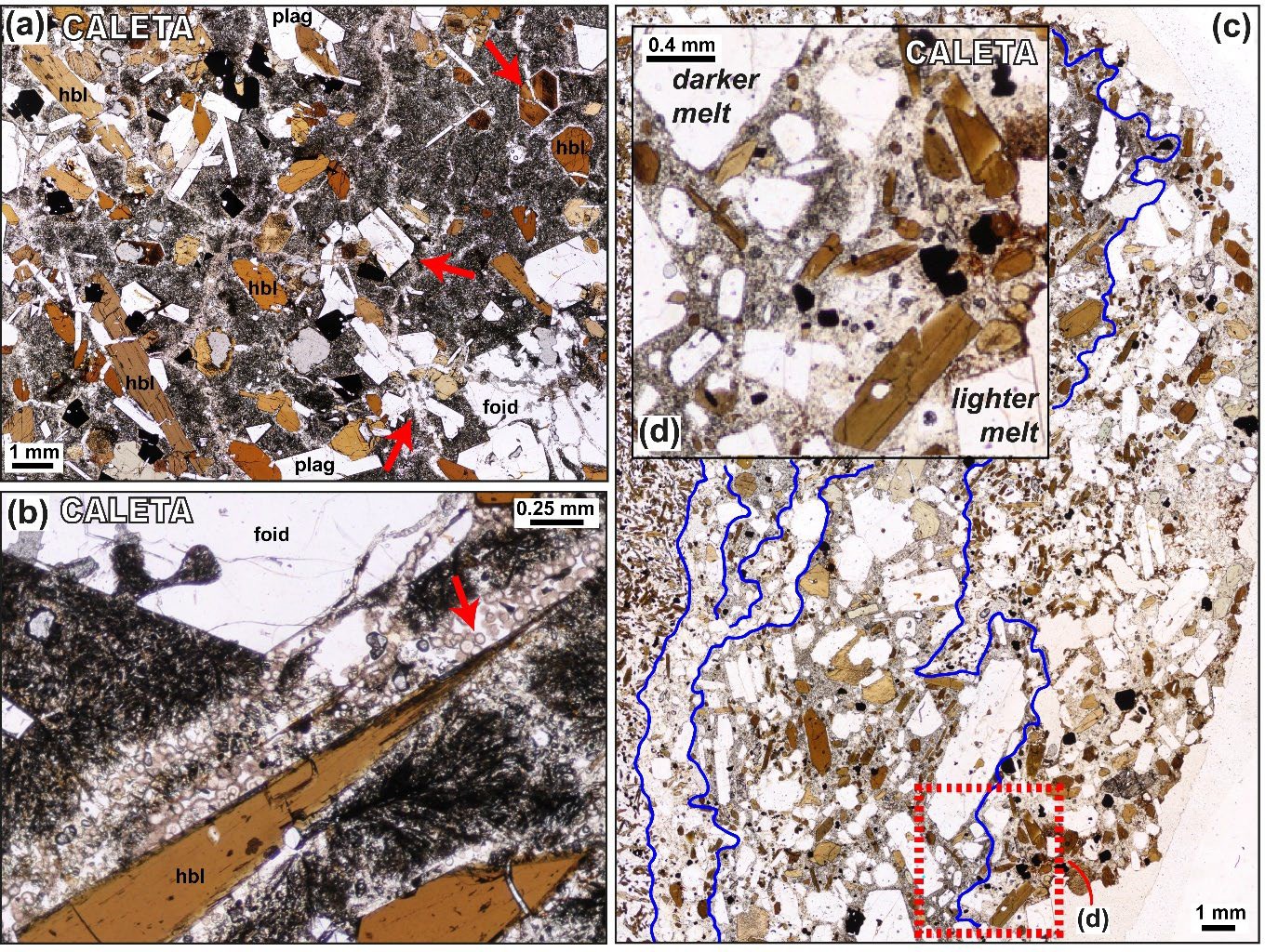


Supplementary Figure 1: Textures in melt-rich regions from Caleta juvenile nodule samples. (A-B) Sample L2-94. Glassy groundmass, cracks cross-cutting minerals (marked in red arrows). (B) Close up of L2-94, bubble textures in cracks, lighter in colour. (C-D) A melt-rich outer layer from sample L2-58 in PPL; blue lines mark the approximate boundaries between a darker more-mafic microcrystalline melt and a lighter, cream coloured more evolved microcrystalline melt. Insert (D) close up of the melt contact


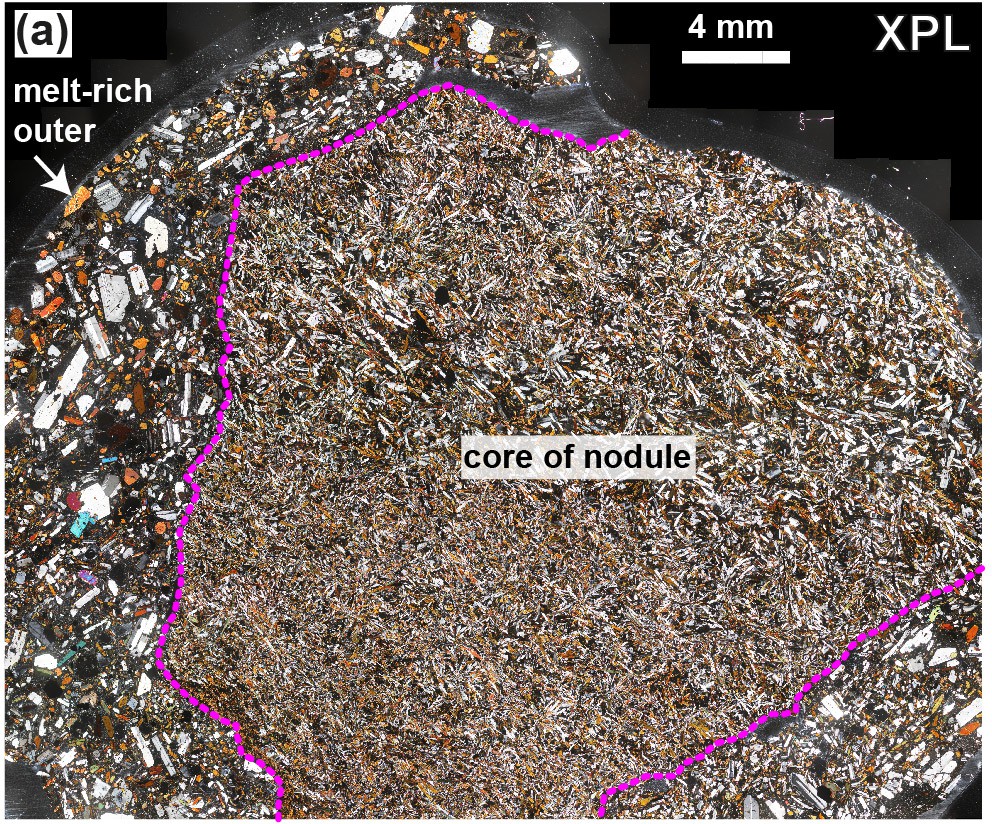


Supplementary Figure 2: Melt-rich coating surrounding core of nodule (Sample L2-58). Two distinct phases in the same sample: the finer-grained xenolith core of the nodule and the melt-rich coarser outer of the sample. Boundary is marked with a purple dashed line.


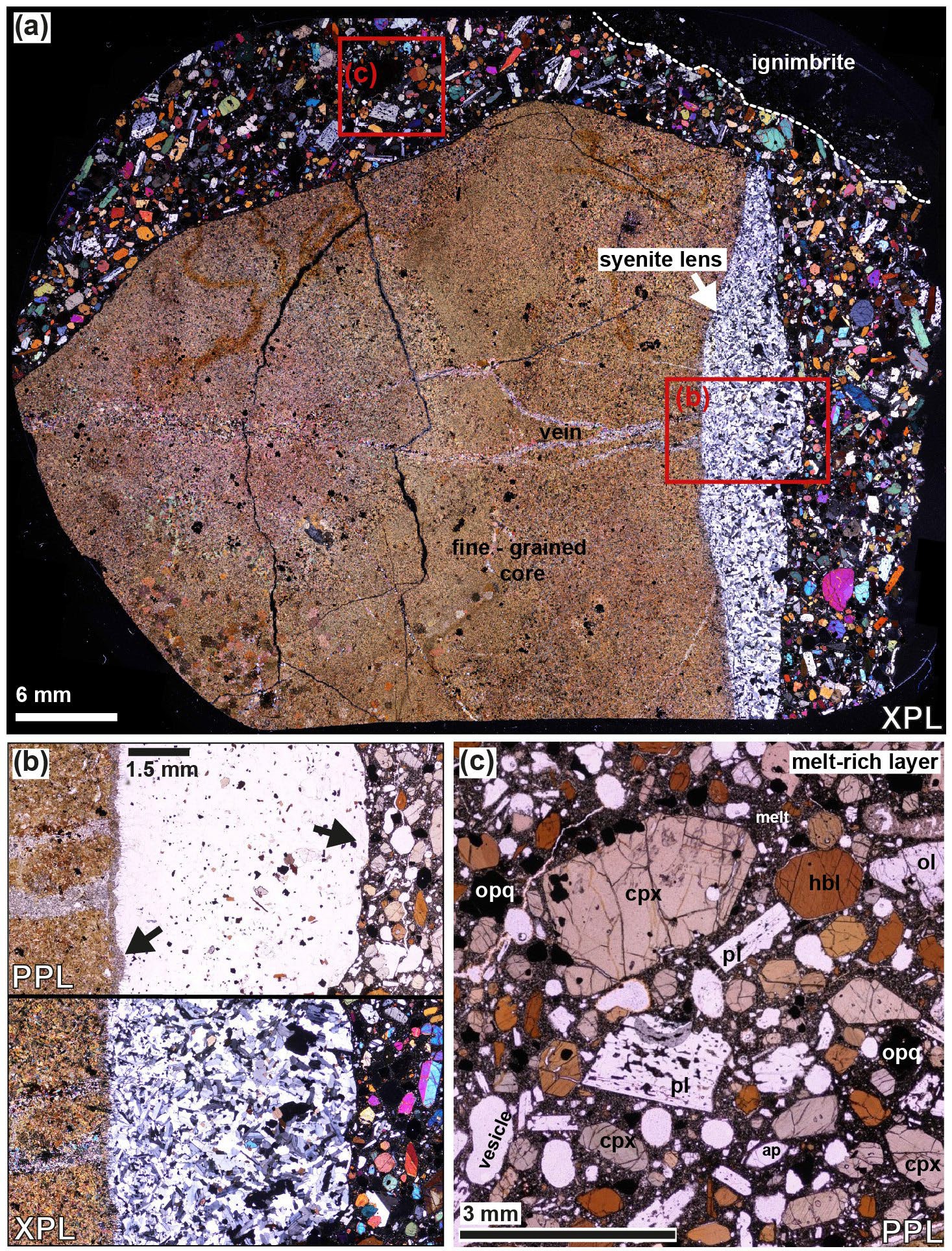


Supplementary Figure 3: (A). Three-phase sample TEM08A: split into fine grained xenolith core, syenite lens and outer juvenile coating. (B). Close up section of syenite lens, arrows in PPL image highlight the contacts between the other lithologies. (C). PPL insert of melt-rich region surrounding and coating the sample.


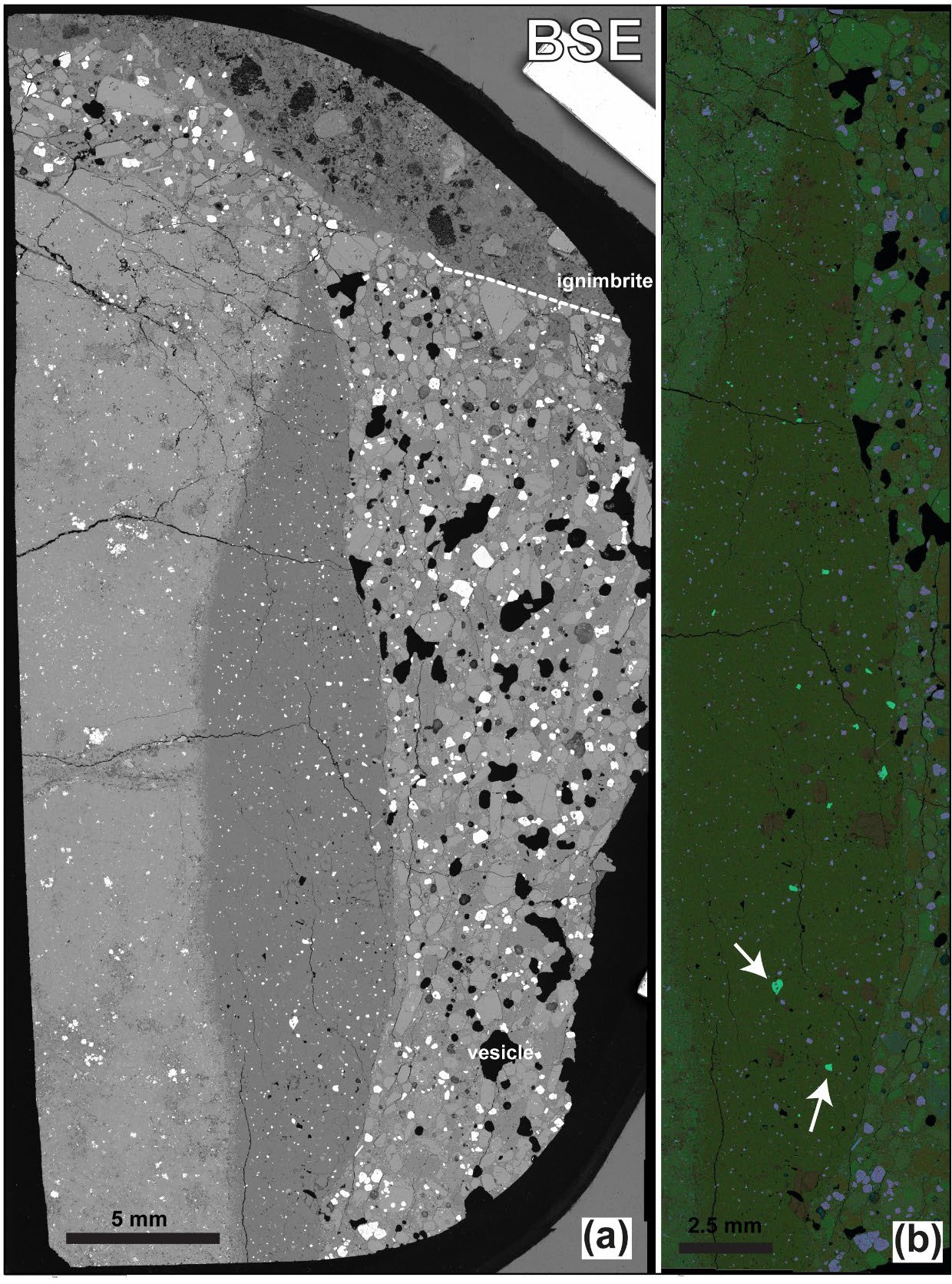


Supplementary Figure 4: Sample TEM-08A, three phase nodule (A): BSE (Backscatter Electron Image) map of TEM-08A; minerals with brighter white colours have heavier molecular weights. (B): Layered elemental map of the syenite lens, Zr concentration has been mapped and coloured bright green to map distribution of zircons in the lens, zircons highlighted with white arrows.

# SEM sites


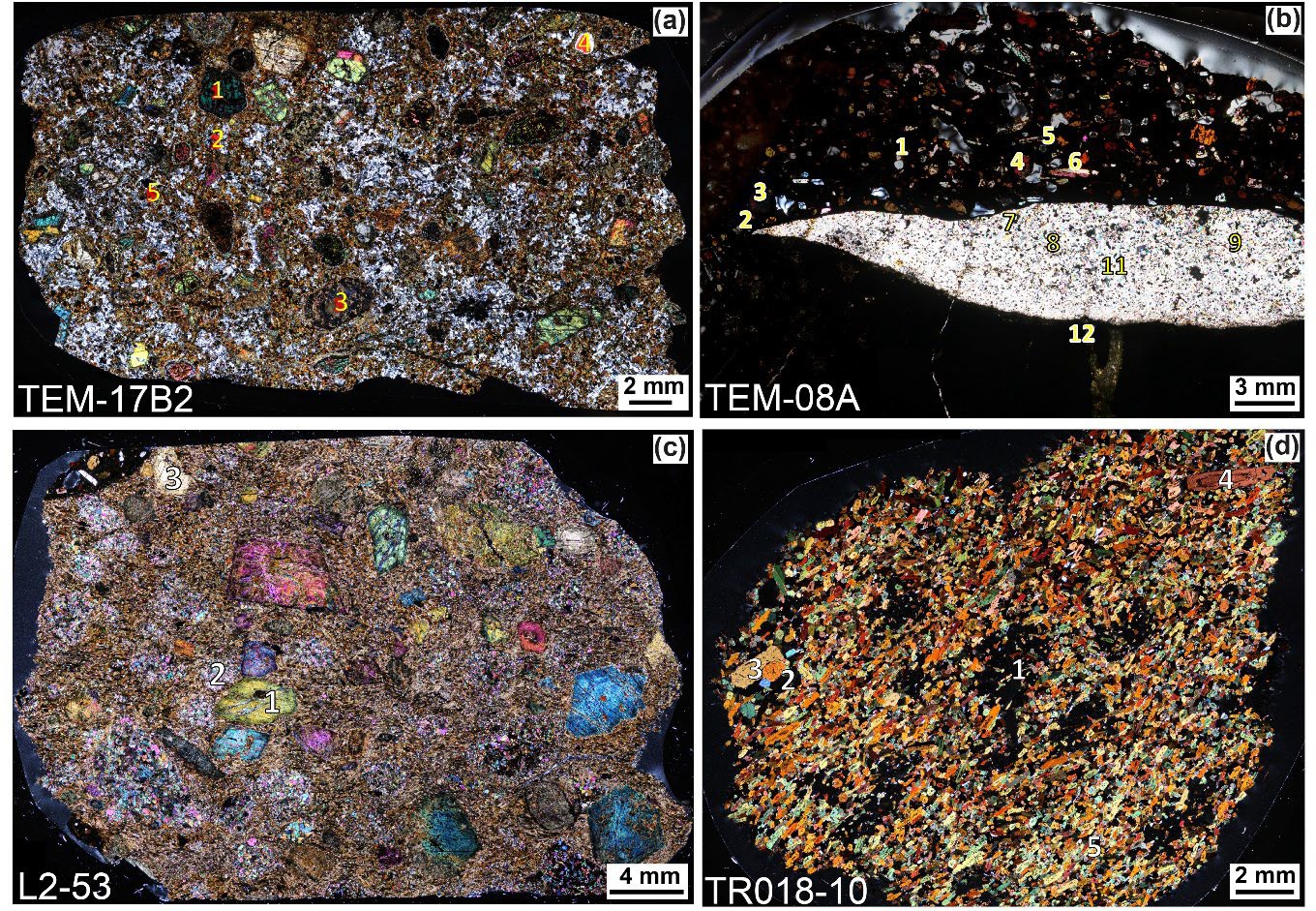

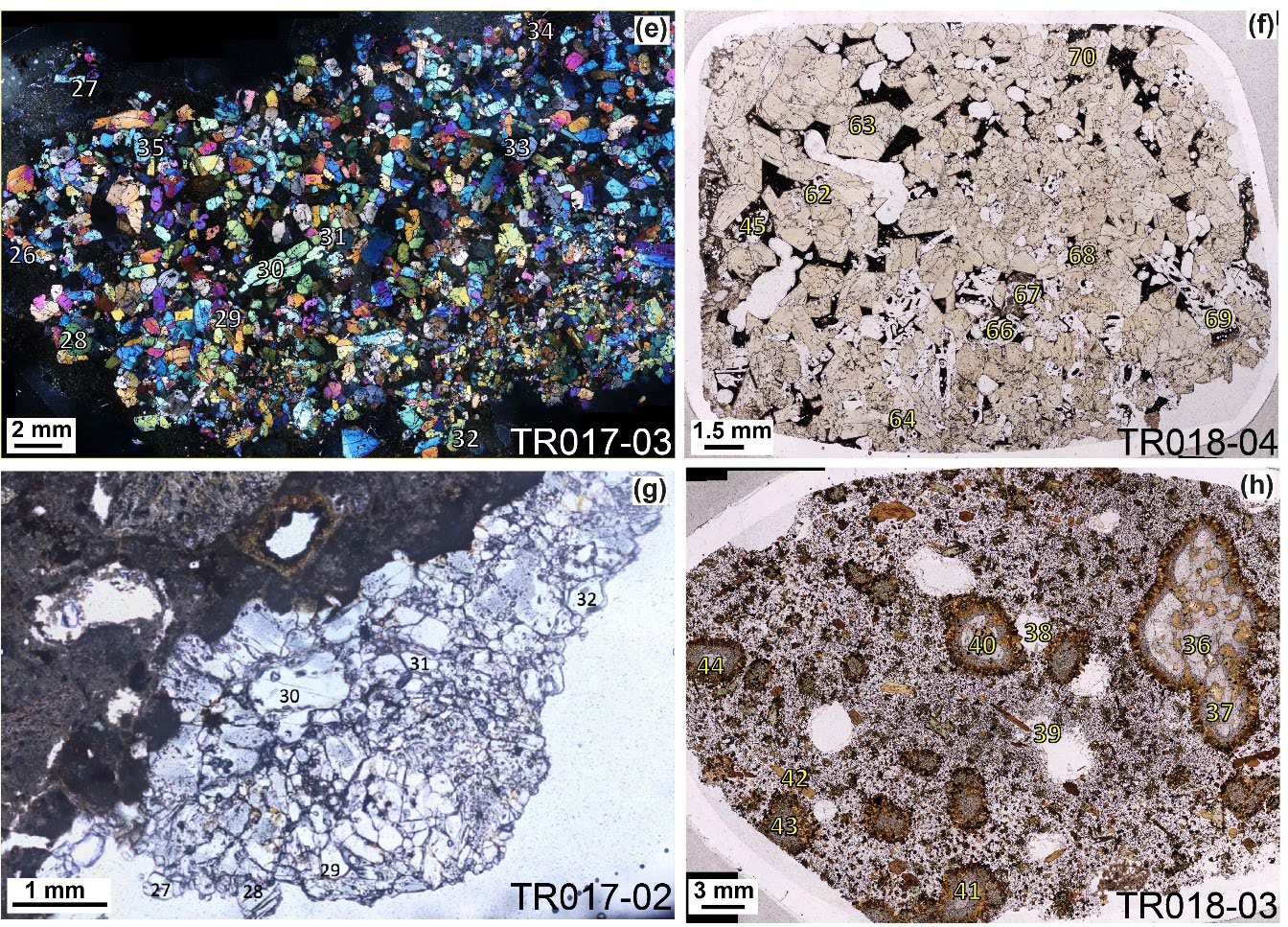


Supplementary Figure 5: Positions of SEM mineral analysis sites and sample ID’s. Samples L2-53, TEM-17B2 and TR018-03 (Caleta) are xenolith plutonic clasts and are distinctly different to juvenile nodules, often containing a high proportion of biotite (A) TEM-17B2; site 1 (ol), site 2 (groundmass phases), site 3 (cpx), site 4 (cpx and groundmass phases) and site 5 (recrystallized ol). (B) TEM-08A; melt-rich outer - site 1 (plag), site 2 (cpx), site 3 (opq), site 4 (cpx), site 5 (hbl), site 6 (plag). Syenite

lens – site 7 (mica), site 8 (opq, kfs), site 9 (zircon), site 11 (zircon, kfs, mica). (C) L2-53, site 1 (cpx), site 2 (ol and mica), site 3 (cpx, mica and groundmass). (D) TR018-10; site 1 (foid), site 2 (foid), site 3 (cpx), site 4 (cpx), site 5 (hbl). (E) TR017-03; site 26-27, 29, 31-32 (cpx), sites 28, 30, 33-35 (ol). (F)TR018-04; sites 45, 62, 65-67, 69 (ol), sites 63-64, 68, 70 (cpx). (G) TR017-02; sites 27-31 (ol), sites 28-32 (cpx). (H) TR018-03; site 37 (ol), site 38 (cpx), site 39 (kfs), site 43 (hbl), site 43 (ol), site 36, 40, 44 (transect through ol plus corona).


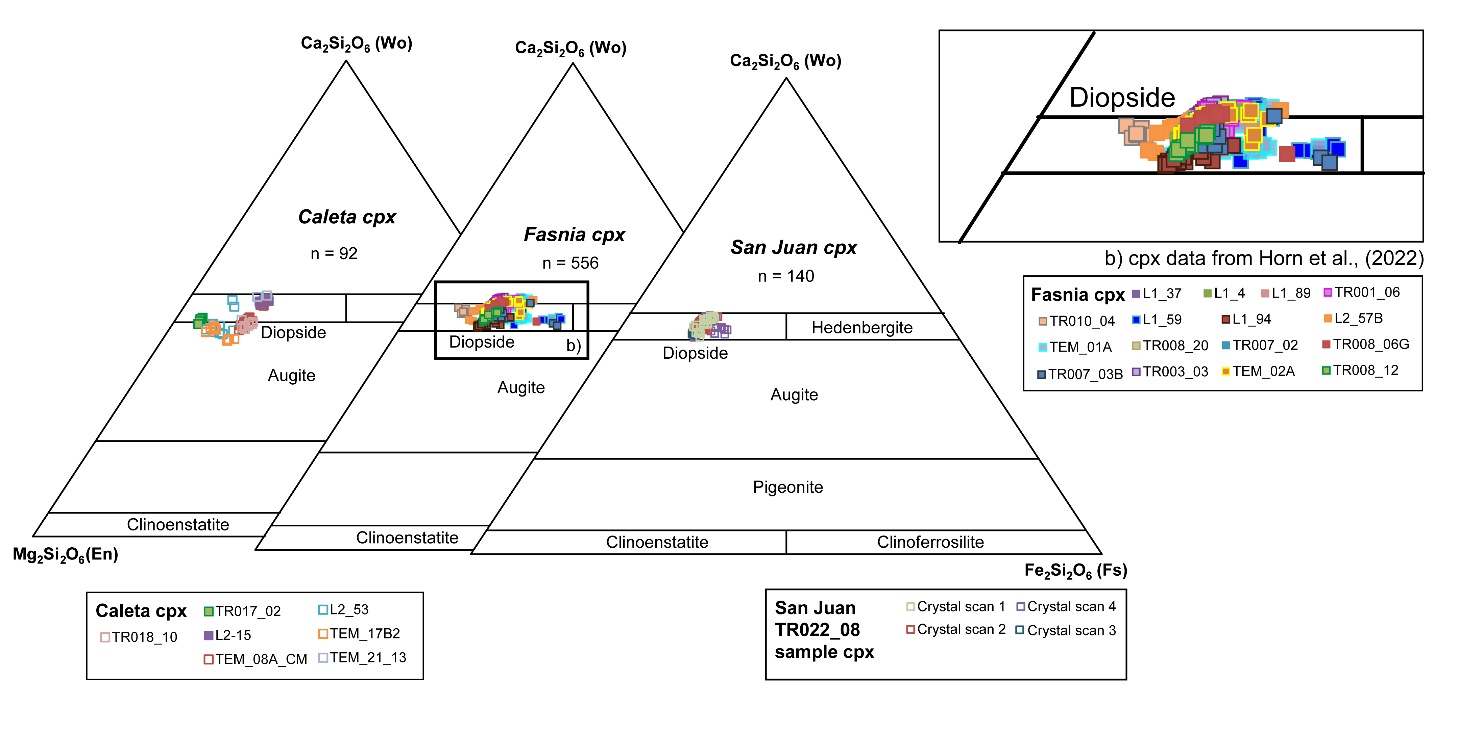


Supplementary Figure 6: Clinopyroxene ternary endmember plots for Caleta, Fasnia and San Juan nodules. EMPA data in filled triangles, SEM data triangle outlines. Pyroxene classification ternary diagram Morimoto (1988) for Ca-Mg-Fe pyroxenes. Fasnia samples (Horn et al., 2022). Data is separated by analysis method; SEM data is plotted as open squares, EMPA data as filled squares. Data from this study and Bromhead (2013).


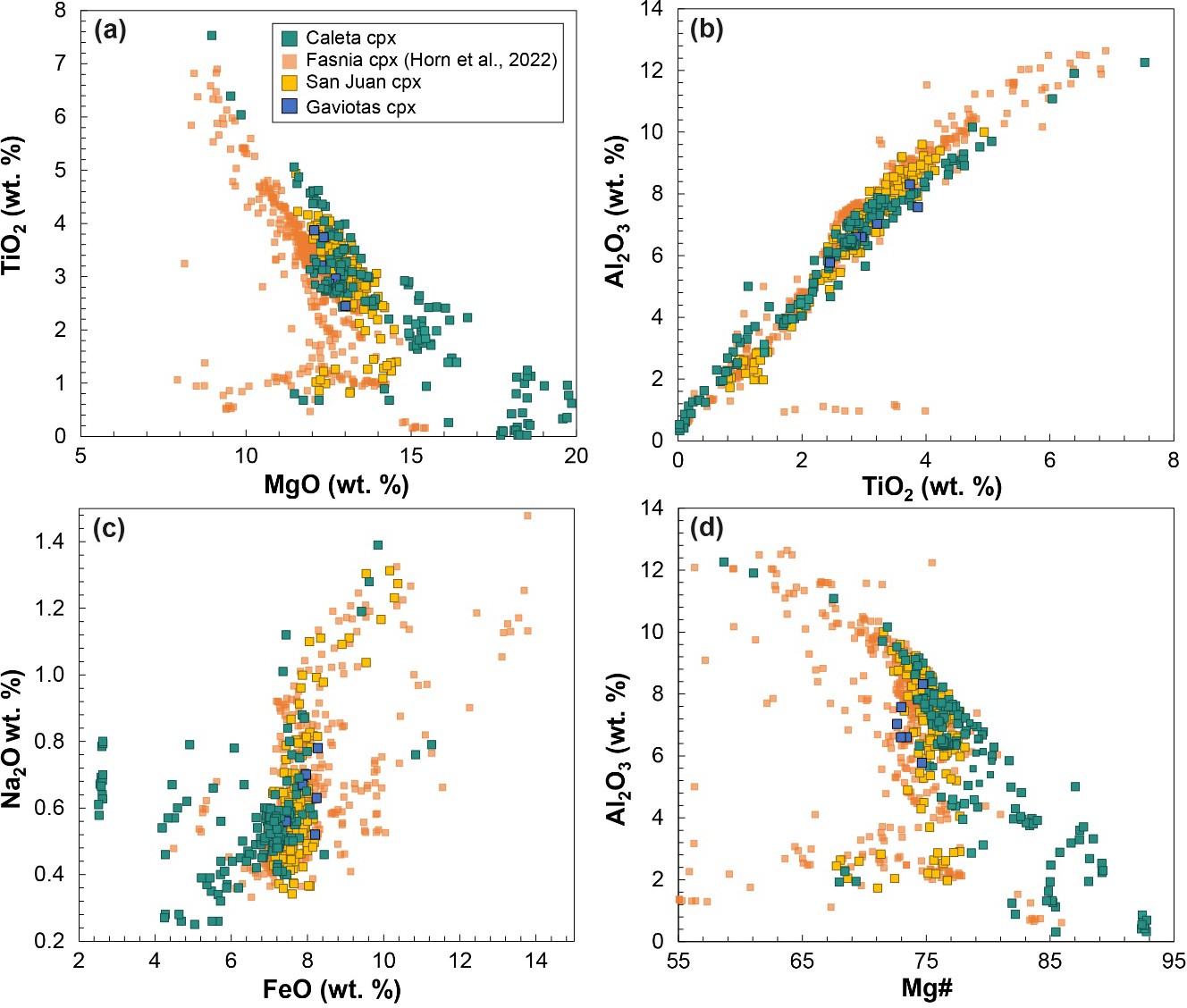


Supplementary Figure 7: Clinopyroxene chemistry of Tenerife nodules. Caleta clinopyroxenes (green squares) include both the juvenile nodules and xenolith clasts sampled. San Juan clinopyroxenes are plotted as yellow squares and Gaviotas as blue squares. A full explanation of the Fasnia Member (orange squares) clinopyroxenes compositions and types is detailed in Horn et al. (2022). Mg# = [atomic Mg/(Mg+ Fe*)], where Fe* = total ^Fe2+^ + Fe^3+^.


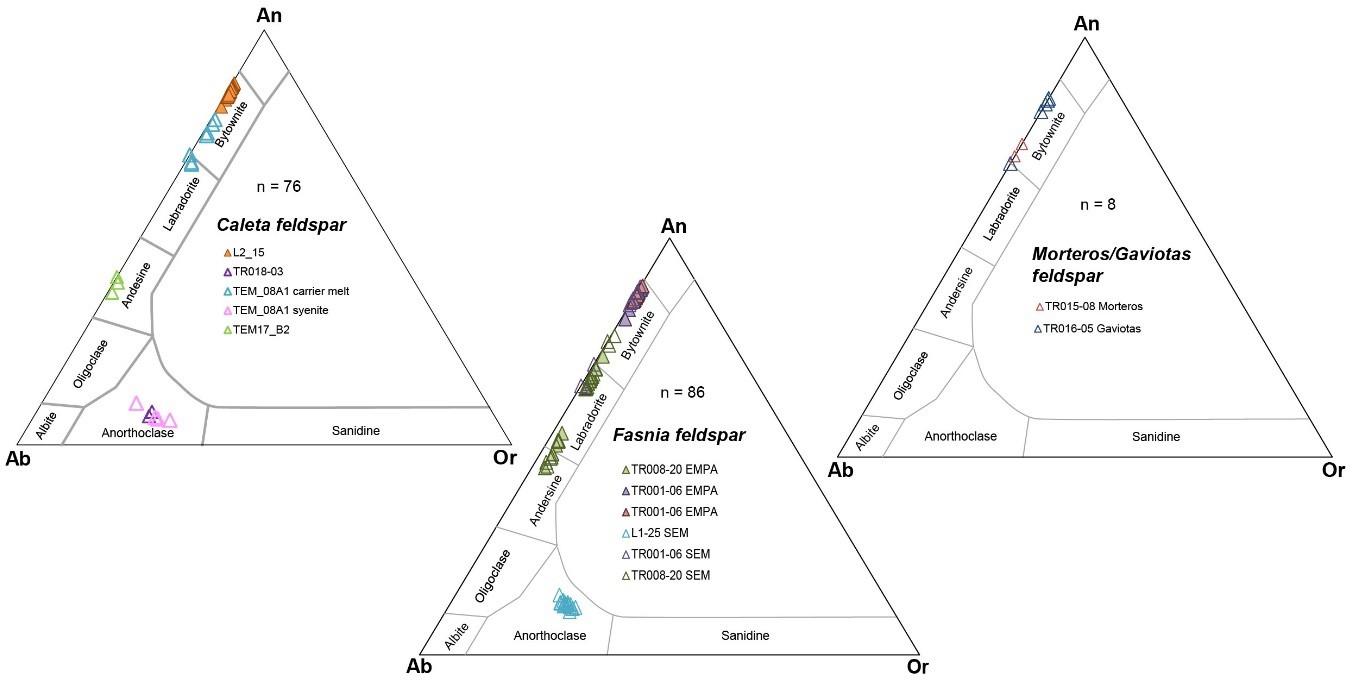


Supplementary Figure 8: Tenerife nodule feldspar plots; a summary of feldspar chemistry. Feldspar classification template from Marshall (1996). Data collected from EMPA is plotted as filled triangles and SEM data as open triangles. Feldspar compositions are expressed in terms of the anorthite (An = Ca/(Ca + Na + K)), albite (Ab = Na/(Ca + Na + K)), and orthoclase (Or = K/(Ca + Na + K)) components, calculated from cations per formula unit normalised to 8 oxygens. (A) Samples TEM08A1 syenite, TEM-17B2 and TR018-03 are in the sub-group of the lithic clasts and are non-juvenile xenoliths.

| *group* | *major phases* | *melt* | *ol* | *cpx* | *pl* | *hbl* | *kfs* | *foid* | *opq* | *apatite* |
| --- | --- | --- | --- | --- | --- | --- | --- | --- | --- | --- |
| 1 | *ol + cpx + opq* | *10* | *24* | *62* |  |  |  |  | *4* |  |
| 2 | *cpx + opq* | *22* |  | *61* |  |  |  |  | *17* |  |
| 3 | *cpx + pl + opq* | *22* |  | *45* | *20* |  |  |  | *12* |  |
| 4 | *cpx + pl + hbl + opq* | *24* |  | *16* | *22* | *27* |  |  | *8* | *3* |
| 5 | *cpx + pl + kfs (± hbl) + opq* | *27* |  | *20* | *20* | *17* | *7* |  | *7* | *2* |
| 6 | *cpx + foid (± pl, kfs, hbl) + opq* | *22* |  | *10* | *18* | *19* | *15* | *7* | *7* | *3* |

Supplementary Table 1: Averaged modal abundance data for 190 Tenerife juvenile nodules, split by major mineral phase assemblage (plotted in Figure 13).
